# Deep rooting in bread wheat is more strongly associated with genotypes than drought stress: evidence from Van Lake Basin genotypes

**DOI:** 10.3389/fpls.2026.1887215

**Published:** 2026-07-22

**Authors:** Burak Özdemir, Harun Bektas, Mehmet Ülker

**Affiliations:** 1 Department of Field Crops, Faculty of Agriculture, Van Yüzüncü Yıl University, Van, Türkiye; 2 Department of Agricultural Biotechnology, Faculty of Agriculture, Siirt University, Siirt, Türkiye

**Keywords:** bread wheat, deep rooting, drought stress, genotypic variation, landraces, root system architecture

## Abstract

Drought stress is a major constraint limiting bread wheat productivity in semi-arid regions, where improved root system architecture is increasingly recognized as a key component of climate resilience. However, it remains unclear whether deep rooting is primarily a genetically determined trait or a plastic response induced by drought stress. This study tested the hypothesis that genotypic background exerts a stronger influence on deep rooting patterns than water deficit itself in bread wheat. Nine genotypes, including seven locally adapted landraces from the Van Lake Basin and two standard cultivars, were grown under controlled greenhouse conditions under well-watered and drought treatments, and root distribution was quantified at three soil depths (0–30, 30–60, and 60–100 cm). Deep root index was not significantly affected by water treatment alone, whereas genotype and genotype × water interaction effects were highly significant, indicating a strong genotype-dependent pattern of root depth allocation. Genotypes showed contrasting drought responses: Bitlis 1–4 increased deep root allocation from 39.8% to 53.1% under drought, while Bitlis 9–1 declined from 44.3% to 32.1%. Associations between root traits and grain yield were weak, suggesting that root system size alone is insufficient to explain drought adaptation. Overall, the results demonstrate that deep rooting in bread wheat is predominantly genotype-dependent rather than uniformly drought-induced. These findings identify promising germplasm with genotype-specific root responses under drought from the Van Lake Basin and provide a basis for integrating root distribution traits into breeding programs aimed at improving wheat performance under water-limited environments.

## 1 Introduction

Wheat (*Triticum aestivum* L.) is one of the most important staple crops worldwide and plays a central role in global food security. In semi-arid regions, however, wheat production is increasingly constrained by drought stress, which limits plant growth, yield formation, and yield stability. Under these conditions, improving yield stability rather than maximizing yield has become a primary objective of modern wheat breeding programs. Nevertheless, progress in enhancing drought tolerance remains limited, largely due to the complex and multi-dimensional nature of plant responses to water deficit (Sallam et al., 2019; Ober et al., 2021).

Among the mechanisms underlying drought adaptation, the root system is widely recognized as a key component of plant performance under water-limited conditions. Root traits such as total root length, root surface area, root volume, and root length density (RLD) directly influence the plant’s capacity to acquire water and nutrients. Recent studies have emphasized that, beyond root system size, root architecture and plasticity are critical for efficient resource capture in heterogeneous soil environments (Schneider and Lynch, 2020; Tracy et al., 2020). Soil water availability is inherently stratified, particularly under drought conditions, where deeper soil layers often represent a more stable and reliable source of water. Although deep rooting has frequently been associated with improved drought tolerance, whether deep rooting reflects an adaptive plastic response to drought stress or an inherent genotypic characteristic remains a question that has received comparatively limited attention yet has fundamental implications for breeding (Wasson et al., 2012; Manschadi et al., 2006). Recent evidence indicates that genotypic identity strongly influences root morphology under drought, with constitutive deep rooting observed in certain genotypes independently of water availability (Frantová et al., 2025).

Genetic variation is a fundamental component of drought adaptation. Locally adapted wheat landraces, which have evolved through long-term farmer selection under marginal conditions, represent valuable genetic resources for improving stress tolerance and yield stability. These genotypes are often characterized by high phenotypic variability and adaptive plasticity, enabling them to maintain performance under fluctuating environments (Dwivedi et al., 2016; Lopes et al., 2015). Root architecture traits in wheat have been shown to be under strong genetic control, with heritability estimates reaching up to 0.99 for certain root angle and depth-related traits, underscoring the potential for targeted selection (Sallam et al., 2024). The Van Lake Basin in eastern Türkiye is recognized as an important micro-center of wheat diversity, where heterogeneous landrace populations have been cultivated for centuries under harsh climatic and edaphic conditions. Despite their potential, the mechanisms underlying drought adaptation in these genotypes — particularly whether deep rooting is a constitutive genetic trait or a drought-triggered plastic response — remain largely unexplored.

A further limitation in existing studies is the tendency to evaluate root responses to drought without distinguishing between genotypic and environmental drivers of root distribution, a distinction that remains a key challenge in the field. Moreover, the weak associations frequently observed between root system size and yield performance suggest that root architecture traits beyond total root length, particularly the spatial deployment of roots within the soil profile, may be more informative for understanding drought adaptation (Palta and Turner, 2019).

Based on these considerations, we hypothesized that (i) deep rooting is primarily a genotype-dependent trait rather than a uniform plastic response to drought stress, (ii) genotypes differ in the direction and magnitude of root distribution changes under drought, reflecting divergent adaptation strategies, and (iii) root system size alone is insufficient to explain yield performance under water-limited conditions, and that genotype-specific root distribution patterns provide additional explanatory value. These hypotheses were evaluated using locally adapted landraces from the Van Lake Basin — a micro-center of wheat diversity shaped by centuries of farmer selection under harsh climatic and edaphic conditions — which represent an underexplored reservoir of adaptive genetic diversity for drought adaptation breeding.

Therefore, the objectives of this study were to (i) evaluate the effects of genotype, water regime, and soil depth on root and shoot traits and quantify their interactions, (ii) determine whether deep rooting is driven primarily by genotypic factors or induced by drought stress, and (iii) assess the relationships between root distribution traits and yield components. By integrating genotypic variation, vertical root distribution, and water stress within a multi-factorial framework, this study aims to advance understanding of drought adaptation mechanisms in wheat and to identify genotype-specific root traits relevant for breeding programs targeting semi-arid environments.

## 2 Materials and methods

### 2.1 Experimental site and plant material

The experiment was conducted under controlled greenhouse conditions at the Faculty of Agriculture, Van Yüzüncü Yıl University (Van, Türkiye). During the experimental period, air temperature was maintained at 20–25 °C and relative humidity ranged between 50–70%. Supplemental LED lighting was provided to maintain a 16 h light/8 h dark photoperiod, with a light intensity of approximately 400–500 µmol m^−2^ s^−1^. Environmental conditions were regulated using an automated greenhouse control system.

A total of nine bread wheat (*Triticum aestivum* L.) genotypes were used in this study (**Table 1**). The seven landrace genotypes included in this study were selected from a larger collection of locally cultivated wheat populations assembled from the Van Lake Basin region as part of a long-term research program focusing on the characterization and conservation of local wheat germplasm (Altuner et al., 2025; Anagun et al., 2025). Selection was based on field observations of key agronomic and morphological traits including heading date, biomass production, chlorophyll retention, and grain yield with the aim of capturing contrasting phenotypic profiles within the collection, including both high- and low-performing genotypes. The genotype designations reflect the district of origin within the Van Lake Basin. Two widely grown standard cultivars were included as reference genotypes to benchmark the landraces against germplasm with documented regional performance: Gerek-79, obtained from the Transitional Zone Agricultural Research Institute, is a widely cultivated cultivar in Central Anatolia known for stable yield under variable rainfall (Bagci et al., 2007), while Karasu-90, obtained from the Eastern Anatolia Agricultural Research Institute, is broadly grown across the Eastern Anatolian highlands and has been characterized as a very hardy, rainfed-adapted cultivar well suited to the cold, semi-arid conditions characteristic of the Van Lake Basin (Olgun et al., 2005). Including both cultivars allowed the relative drought and root performance of the landraces to be evaluated against modern reference material from contrasting breeding backgrounds.

**Table 1:** Bread wheat genotypes used in the study, their classification, and origin.

Genotype	Type	Origin
Bitlis 1-4	Landrace	Van Lake Basin (Project FBA-2019-8276)
Bitlis 9-1	Landrace	Van Lake Basin (Project FBA-2019-8276)
Erciş 5-6	Landrace	Van Lake Basin (Project FBA-2019-8276)
Erciş 8-2	Landrace	Van Lake Basin (Project FBA-2019-8276)
Muradiye 12-2	Landrace	Van Lake Basin (Project FBA-2019-8276)
Patnos 1-4	Landrace	Van Lake Basin (Project FBA-2019-8276)
Saray 2-5	Landrace	Van Lake Basin (Project FBA-2019-8276)
Gerek-79	Standard cultivar	Transitional Zone Agricultural Research Institute
Karasu-90	Standard cultivar	Eastern Anatolia Agricultural Research Institute

### 2.2 Experimental design

The experiment was arranged as a factorial design within a randomized complete block design (RCBD) with five replications. The experimental factors were genotype (nine levels) and water regime (well-watered and drought conditions). Each PVC tube was considered an individual experimental unit.

The study was conducted in two independent growing seasons: the first was established in September 2021 (autumn growing season) and the second in March 2022 (spring growing season). Growing season was included as a fixed factor in the statistical model, as the two seasons represented distinct environmental periods with specific photoperiod and temperature profiles that were of direct interest in this study. Both growing seasons followed identical experimental protocols, including vernalization treatment, irrigation management, and harvest procedures, ensuring comparability between seasons.

Seeds were vernalized at 2 °C for 5 weeks prior to sowing in both growing seasons. Three seeds were sown per tube, and seedlings were thinned to one plant per tube after emergence.

### 2.3 Growth medium and tube system

The experiment was conducted in PVC tubes (1 m in length and 10 cm in diameter). Polyethylene liners were placed inside each tube prior to filling to facilitate intact root extraction at harvest, following established deep root phenotyping protocols (Manschadi et al., 2006).

The growth medium consisted of a mixture of sieved soil (5 mm mesh) and washed sand at a ratio of 3:1 (v/v). The soil mixture had a pH of 8.2, electrical conductivity of 0.25 dS m^−1^, 10.5% CaCO_3_, 1.13% organic matter, 0.06% total nitrogen, and 5.50 mg kg^−1^ available phosphorus, with a sandy loam texture. Soil physicochemical properties were determined using standard laboratory procedures. The soil was packed uniformly in layers, with the mass of soil mixture added per layer calculated from tube volume to achieve a target bulk density of approximately 1.40 g cm^−3^, ensuring consistent bulk density and water distribution throughout the soil profile.

### 2.4 Irrigation and fertilization

Field capacity (FC) of the soil mixture was determined gravimetrically prior to the experiment by saturating soil columns and allowing free drainage until constant weight was achieved. The air-dry weight of each tube was recorded individually before filling to enable precise calculation of soil water content throughout the experiment.

Soil moisture monitoring was initially performed using time-domain reflectometry (TDR) probes inserted at 20, 40, 60, and 80 cm depth intervals in each tube. However, repeated measurements through the same access points resulted in localized void formation around the probe insertion sites, compromising measurement accuracy. Consequently, a gravimetric monitoring approach was adopted as the primary method for soil moisture determination. Tubes were weighed every 2–3 days using a precision balance, and soil water content was calculated from the difference between current tube weight and the known air dry weight. This gravimetric approach ensured reliable and consistent moisture control throughout the experimental period, and TDR readings were used periodically for cross-validation during the early stages of the experiment.

All plants were maintained at approximately 90% FC from sowing until the onset of stem elongation, corresponding to the wet establishment phase typical of early spring growth in the Van Lake Basin, where winter snowmelt and spring precipitation keep topsoil moisture high during seedling establishment. Drought stress was subsequently imposed in two successive stages to mimic the progressive, terminal-type drought pattern characteristic of the region’s cold semi-arid continental climate (Köppen Dsb), in which precipitation declines sharply through late spring and summer as snowmelt inputs diminish, coinciding with the stem elongation through grain-filling period: soil moisture was reduced from 90% to 50% FC over a 10-day transition period beginning at stem elongation and maintained at 50% FC for approximately 25 days until the milk stage, after which it was further reduced from 50% to 25% FC over a 7-day transition period and maintained at 25% FC for approximately 20 days until physiological maturity. These target levels and transition periods were based on staged dry-down protocols used in previous wheat root-phenotyping studies (Manschadi et al., 2006) and were intended to impose progressively intensifying water deficit during the reproductive and grain-filling phases, when drought has the greatest impact on yield. Control plants were maintained at approximately 90% FC throughout the entire growth period. The required amount of water was calculated for each tube individually based on its recorded air dry weight and target FC level, and added accordingly at each monitoring interval.

Fertilization was applied in three stages: 4 kg da−1 N and 4 kg da^−1^ P (20–20–0 compound fertilizer) at sowing, 7.5 kg da^−1^ N (urea, 46% N) at the tillering stage, and 4 kg da^−1^ N (ammonium nitrate, 33% N) at the stem elongation stage.

### 2.5 Root sampling and measurements

At physiological maturity, aboveground biomass was harvested and root systems were extracted by removing the polyethylene liners from the PVC tubes. Roots were carefully separated from the soil and gently washed under running water to remove adhering particles, with particular care taken to minimize the loss of fine roots.

The cleaned root systems were divided into three depth intervals: 0–30 cm, 30–60 cm, and 60–100 cm. Root samples from each depth interval were placed in a transparent water-filled tray and scanned using a flatbed scanner (Plustek OpticPro PR320) at a resolution of 600 dpi. A transparent plate was used to ensure uniform root spreading and to minimize overlap during scanning. Root samples were kept submerged in water between washing and scanning to prevent drying and structural distortion.

Images were analyzed using RhizoVision Explorer software (Seethepalli et al., 2021) under consistent threshold settings applied uniformly across all samples. The following root traits were determined for each depth interval: total root length, root surface area, root volume, average root diameter, root length density (RLD), specific root length (SRL), and root tissue density (RTD).

Root length density (RLD) was calculated as total root length divided by the soil volume of the corresponding depth interval. The deep root index (DRI) was calculated as the proportion of root length in the deepest soil layer (60–100 cm) relative to total root length, expressed as a percentage:


$$\text{DRI}\left(\%\right)=\left({\text{Root length}}_{60-100}/\text{Total root length}\right)\times 100$$

Because the deepest sampling interval (60–100 cm) was 10 cm thicker than the two upper intervals (0–30 and 30–60 cm), DRI reflects the proportion of total root length recovered from this predefined deepest interval rather than a thickness-normalized measure of root distribution. As the same depth scheme was applied uniformly across all genotypes and water treatments, comparisons among genotypes and treatments remain directly comparable within the common sampling scheme; depth-normalized comparisons are additionally provided through RLD (**Table 2**; **Supplementary Table 2**).

**Table 2:** Analysis of variance (F values) for depth-dependent root traits under different genotypes and water treatments.

Source	df	Root length	Surface area	RLD
GS	1	210.485***	39.751***	221.958***
G	8	82.168***	68.897***	83.965***
W	1	131.100***	165.742***	148.741***
D	2	765.630***	2128.385***	648.861***
G×W	8	24.336***	10.715***	21.847***
G×D	16	17.597***	12.240***	15.029***
W×D	2	17.420***	39.707***	24.749***
G×W×D	16	21.819***	12.245***	19.871***
CV (%)	—	14.9	16.1	14.1

GS, Growing Season; G, Genotype; W, Water treatment; D, Soil depth. ns, not significant; *p < 0.05; **p < 0.01; ***p < 0.001. Detailed ANOVA results for all root traits are presented in **Supplementary Table 2**.

### 2.6 Shoot measurements

At harvest, grain yield, biological yield, and harvest index were determined after oven-drying plant samples at 65 °C to constant weight. SPAD chlorophyll content was measured at the milk stage using a portable chlorophyll meter (SPAD-502, Konica Minolta), with three readings taken per plant and averaged. Additional morphological and physiological traits, including plant height, spike length, tiller number, heading date, and maturity date, were recorded and are presented in the **Supplementary Materials** (**Supplementary Table 4**).

### 2.7 Statistical analysis

All statistical analyses were performed using R software (version 4.2.0; R Core Team, 2022). The statistical model included genotype (G), water treatment (W), soil depth (D), and their two- and three-way interactions (G×W, G×D, W×D, and G×W×D). Growing season was included as a fixed factor in the statistical model, as the two seasons represented distinct environmental periods with specific photoperiod and temperature profiles that were of direct interest in this study.

Analysis of variance (ANOVA) was conducted using the “lme4” package (Bates et al., 2015). Mean comparisons were performed using Tukey’s honestly significant difference (HSD) test at p < 0.05. Significance levels are indicated as follows: ns = not significant; * p < 0.05; ** p < 0.01; *** p < 0.001. Prior to analysis, data were tested for normality and homogeneity of variance using the Shapiro–Wilk and Levene tests, respectively. Most traits satisfied both assumptions and were analyzed without transformation. Where normality or homogeneity of variance was not met (root surface area, root volume, and specific root length), a log10 transformation was applied prior to ANOVA, which satisfactorily corrected the distributional assumptions; results for these traits are reported on the back-transformed (original) scale for interpretability.

Pearson correlation analysis was used to evaluate relationships among root traits, shoot traits, and yield components. Principal component analysis (PCA) was performed using the “FactoMineR” package (Lê et al., 2008) to identify major sources of variation and trait associations. All figures were generated using the “ggplot2” package (Wickham, 2016). Simple linear regression analyses were performed to examine the relationships between grain yield and root traits (deep root index and total root length) under control and drought conditions separately, using the base R stats package.

## 3 Results

### 3.1 Effects of genotype, water treatment, and growing season on yield and root traits

Grain yield, biological yield, harvest index, total root length, and deep root index were all significantly affected by genotype and growing season (**Table 3**; **Supplementary Table 1**). Water treatment significantly affected all evaluated traits except deep root index. A key finding of this study was that deep root index was not significantly influenced by water treatment alone (F = 0.70, p > 0.05), whereas genotype (F = 28.03, p < 0.001) and genotype × water interaction (F = 41.19, p < 0.001) were highly significant.

**Table 3:** Analysis of variance (F values) for yield-related and selected root traits under different genotypes and water treatments.

Source	df	Grain yield	Biological yield	Harvest index	Root length	Deep root index
Growing Season (GS)	1	1742.70***	2823.11***	68.42***	134.24***	13.06***
Genotype (G)	8	463.85***	200.13***	184.07***	52.40***	28.03***
Water (W)	1	4651.98***	2261.44***	467.70***	83.61***	0.70ns
GS× G	8	59.46***	66.62***	38.61***	25.42***	16.19***
GS × W	1	0.87ns	18.66***	41.61***	12.20***	0.06ns
G × W	8	74.11***	40.24***	59.00***	15.52***	41.19***
GS × G × W	8	4.16***	20.34***	7.37***	5.67***	20.68***
CV (%)		4.31	4.32	4.60	10.79	8.62

GS, Growing Season; G, Genotype; W, Water treatment; df, degrees of freedom; CV (%): coefficient of variation. ns, not significant; *p < 0.05; **p < 0.01; ***p < 0.001. Detailed ANOVA results for all shoot traits are presented in **Supplementary Table 1**.

Among yield-related traits, water treatment exerted the strongest effect on grain yield (F = 4651.98, p < 0.001), followed by biological yield (F = 2261.44, p < 0.001) and harvest index (F = 467.70, p < 0.001), indicating that soil water availability was the primary driver of yield formation. Despite this strong environmental influence, the significant genotype × water interaction for grain yield (F = 74.11, p < 0.001), biological yield (F = 40.24, p < 0.001), and harvest index (F = 59.00, p < 0.001) demonstrated that genotypes responded differently to water limitation.

Total root length was also significantly affected by genotype, water treatment, and their interaction (p < 0.001), indicating contrasting root growth responses under drought conditions across genotypes.

Growing season had a significant effect on all measured traits (**Table 3**), reflecting environmental differences between the two experimental periods. Although air temperature, relative humidity, and supplemental photoperiod were held within the same target ranges in both seasons, the September 2021 (autumn) and March 2022 (spring) sowing dates differed in ambient outdoor daylength and natural light intensity reaching the greenhouse, which the automated control system supplemented but did not fully equalize between seasons. Growing season was therefore included *a priori* as a fixed factor in the statistical model rather than treated as unexplained noise, consistent with the view that the two seasons represented distinct, biologically meaningful environmental periods rather than a confound. Significant growing season × genotype and growing season × water interactions for most traits further indicate that genotype performance and drought responses were influenced by seasonal conditions. Detailed ANOVA results are presented in **Supplementary Table 1**.

### 3.2 Genotypic variation in yield and root traits under control and drought conditions

**Figure 1:**
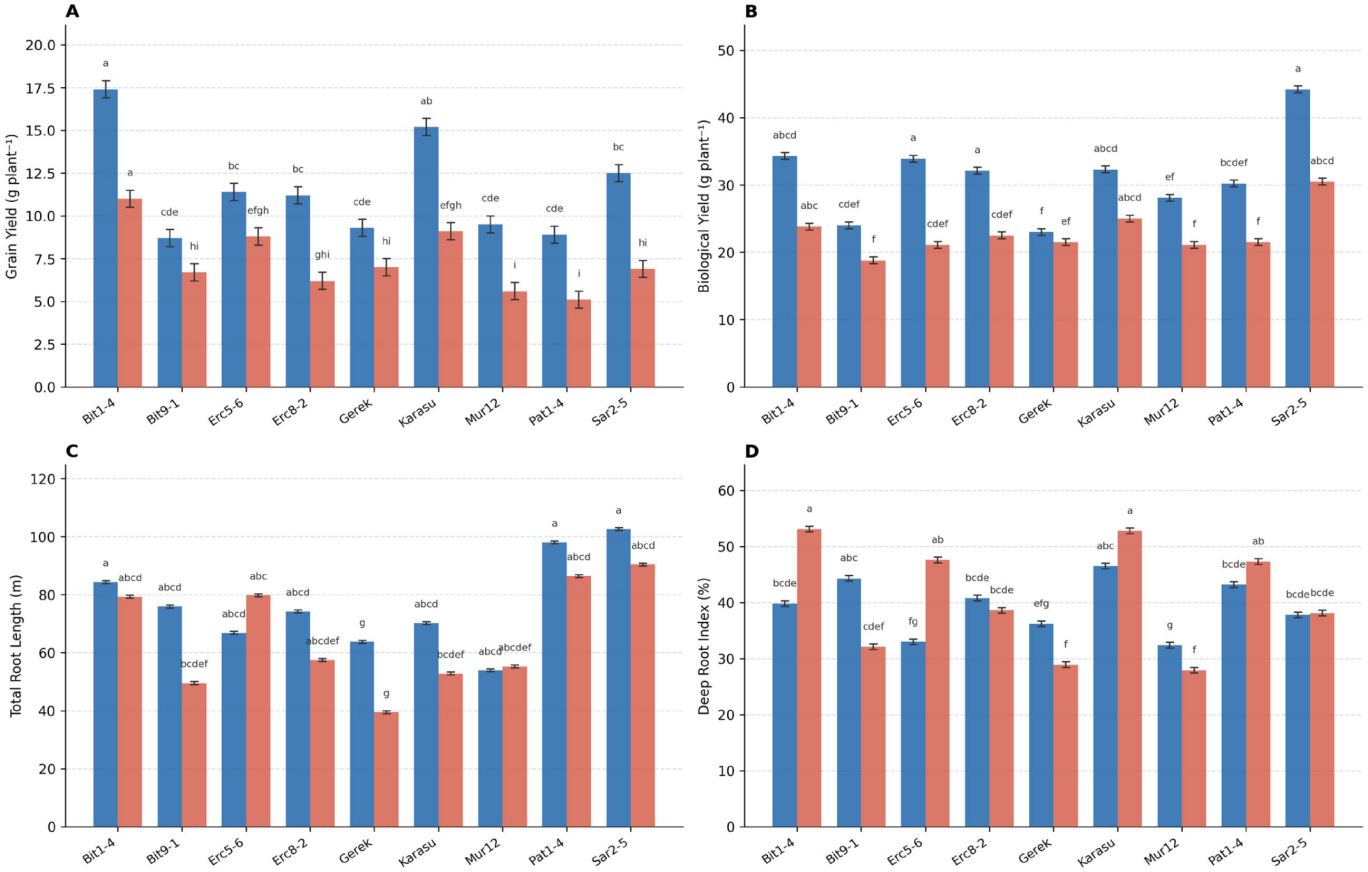
Genotypic variation in **(A)** grain yield, **(B)** biological yield, **(C)** total root length, and **(D)** deep root index under control and drought conditions. Different letters indicate significant differences among genotypes within each treatment according to Tukey’s test (p < 0.05).

Grain yield varied significantly among genotypes under both control and drought conditions (**Figure 1A**). Under control conditions, grain yield ranged from 9.5 g plant^−1^ (Muradiye 12-2) to 17.4 g plant^−1^ (Bitlis 1-4). Drought stress reduced grain yield in all genotypes; however, the magnitude of reduction varied considerably among genotypes, with values ranging from 5.6 g plant^−1^ (Muradiye 12-2) to 11.0 g plant^−1^ (Bitlis 1-4) under drought conditions. Bitlis 1–4 exhibited the highest grain yield under drought, followed by Karasu (9.1 g plant^−1^) and Erciş 5-6 (8.8 g plant^−1^), whereas Muradiye 12–2 and Patnos 1–4 showed the lowest values.

Biological yield followed a similar pattern (**Figure 1B**), with a general decline under drought conditions across all genotypes. Under control conditions, biological yield ranged from 25.6 g plant^−1^ (Gerek) to 42.4 g plant^−1^ (Erciş 8-2), whereas under drought it decreased to values between 20.7 g plant^−1^ (Patnos 1-4) and 29.9 g plant^−1^ (Erciş 8-2). Erciş 8–2 maintained the highest biological yield under both conditions.

Total root length exhibited contrasting responses among genotypes under drought conditions (**Figure 1C**). Under control conditions, root length ranged from 54.9 m (Gerek) to 90.1 m (Saray 2-5). Although most genotypes showed reduced root length under drought, Erciş 5–6 and Karasu exhibited increased values (from 58.2 to 69.4 m and 58.1 to 64.9 m, respectively).

Deep root index also varied among genotypes under both control and drought conditions (**Figure 1D**). Genotypes differed in both the magnitude and direction of change in deep root allocation under drought stress. Bitlis 1–4 increased deep root index from 39.8% to 53.1% (+33.4%) and Erciş 5–6 from 33.0% to 47.6% (+44.2%) under drought conditions. In contrast, Bitlis 9–1 decreased from 44.3% to 32.1% (−27.5%) and Gerek from 41.2% to 28.9% (−29.9%). Karasu showed a moderate increase (from 46.5% to 52.8%, +13.5%), while Saray 2–5 remained largely unchanged (+0.8%).

Overall, substantial genotypic variation was observed in both aboveground and belowground traits, with genotypes exhibiting distinct and contrasting response patterns under drought conditions.

### 3.3 Depth-dependent variation in root traits

Root traits varied significantly across soil depths, with soil depth exerting the largest effect on root length, surface area, and root length density (RLD) among all factors tested (**Table 2**; **Supplementary Table 2**). The F-values for depth were substantially higher than those for genotype and water treatment (F = 765.63 for root length; F = 2128.39 for surface area; F = 648.86 for RLD), indicating that soil depth was the dominant source of variation in root trait distribution. Growing season also had a significant effect on all depth-dependent root traits (F = 210.49 for root length; F = 39.75 for surface area; F = 221.96 for RLD; p < 0.001). Detailed ANOVA results for all root traits are presented in **Supplementary Table 2**.

Across genotypes, root length generally decreased from the upper (0–30 cm) to the middle soil layer (30–60 cm), whereas values in the deepest layer (60–100 cm) varied considerably among genotypes. Under control conditions, root distribution was relatively consistent across soil depths. For example, Bitlis 1–4 exhibited root lengths of 33.30 m, 20.96 m, and 35.79 m across the three depth intervals, with detailed values for all genotypes presented in **Supplementary Table 3**.

Under drought conditions, shifts in root distribution across depths were observed in several genotypes. Bitlis 1–4 increased root length in the deepest layer from 35.79 m under control conditions to 42.84 m under drought, accompanied by an increase in RLD from 1.14 to 1.36 cm cm^−3^. Similarly, Erciş 5–6 showed a marked increase in deep root length from 19.59 m to 34.10 m, with RLD increasing from 0.62 to 1.09 cm cm^−3^.

In contrast, other genotypes showed reduced root length in the deepest soil layer under drought conditions. Bitlis 9–1 decreased from 38.33 m to 17.17 m, and Gerek from 22.92 m to 11.33 m in the 60–100 cm layer.

Root surface area and RLD followed trends similar to those observed for root length across soil depths. Significant G×D, W×D, and G×W×D interactions were observed for all measured traits (**Table 2**), indicating that root distribution patterns varied depending on both genotype and water availability.

Overall, root traits showed clear depth-dependent variation, with genotypes differing markedly in the distribution and magnitude of root growth along the soil profile under both control and drought conditions.

### 3.4 Relationships among yield and root traits

**Figure 2:**
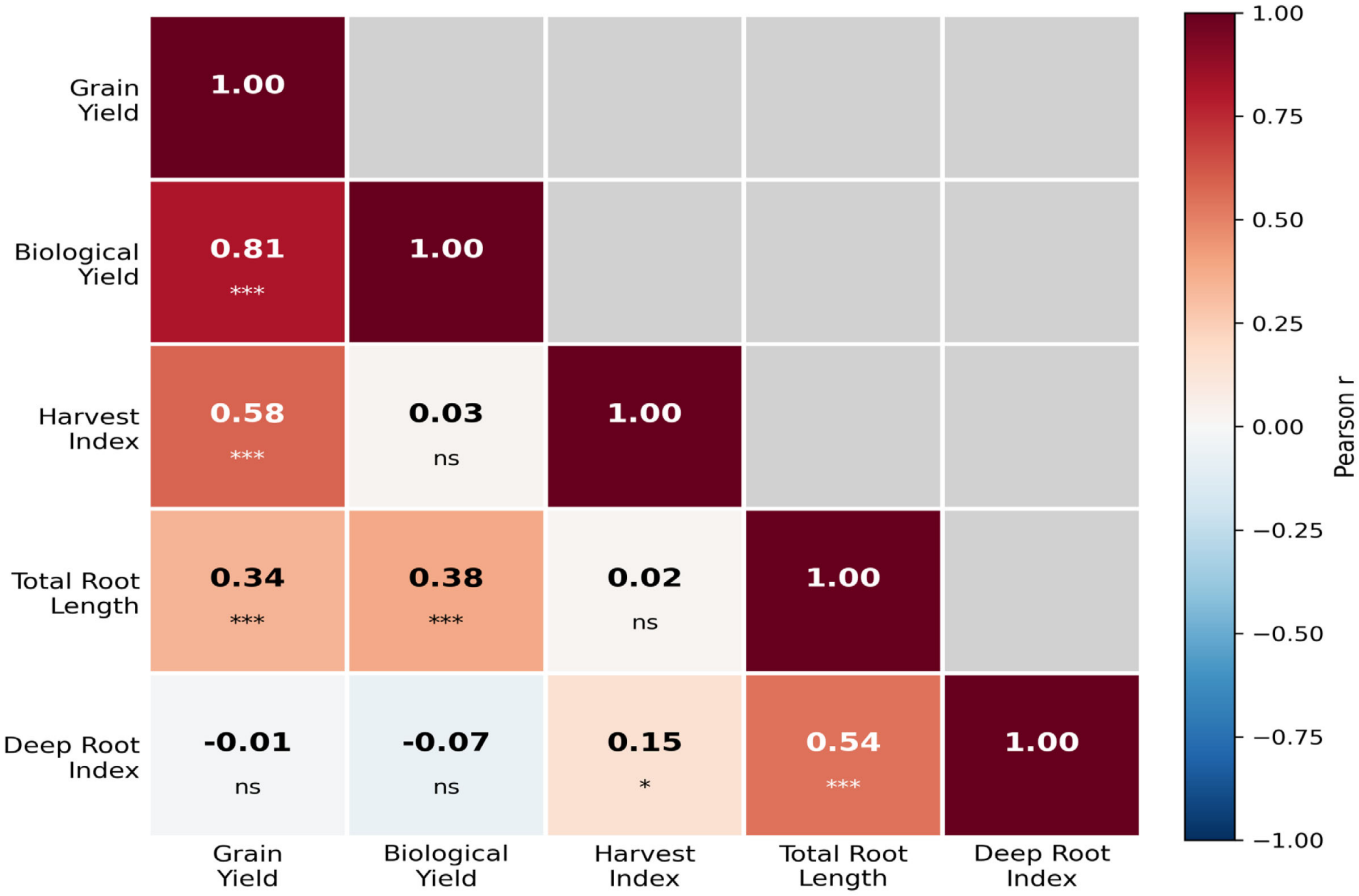
Pearson correlation matrix showing relationships among grain yield, biological yield, harvest index, total root length, and deep root index. The color gradient indicates the magnitude and direction of correlations (red for positive and blue for negative associations). Numerical values represent correlation coefficients (r). Significance levels are indicated as follows: *p < 0.05, **p < 0.01, ***p < 0.001, and ns, non-significant.

Correlation analysis revealed strong positive relationships among yield-related traits (**Figure 2**; **Supplementary Tables 5**, **S6**). Grain yield showed a strong positive correlation with biological yield (r = 0.81, p < 0.001) and moderate positive correlations with harvest index (r = 0.58, p < 0.001) and SPAD chlorophyll content (r = 0.41, p < 0.001).

In contrast, relationships between grain yield and root traits were weak. Total root length showed a low positive correlation with grain yield (r = 0.34, p < 0.001), while deep root index showed no significant correlation with grain yield (r = −0.01, p = 0.894). Similarly, biological yield and harvest index exhibited weak or negligible correlations with root traits.

Among root traits, strong positive correlations were observed between morphologically related variables, particularly between total root length and surface area (r = 0.94, p < 0.001). Total root length was also moderately correlated with deep root index (r = 0.54, p < 0.001).

Overall, correlations among shoot-related traits were considerably stronger than those between shoot and root traits, indicating a degree of independence between aboveground yield performance and belowground root system characteristics. Detailed correlation coefficients for all shoot and root traits are provided in **Supplementary Table 5** and **S6**.

### 3.5 Principal component analysis of trait associations

**Figure 3:**
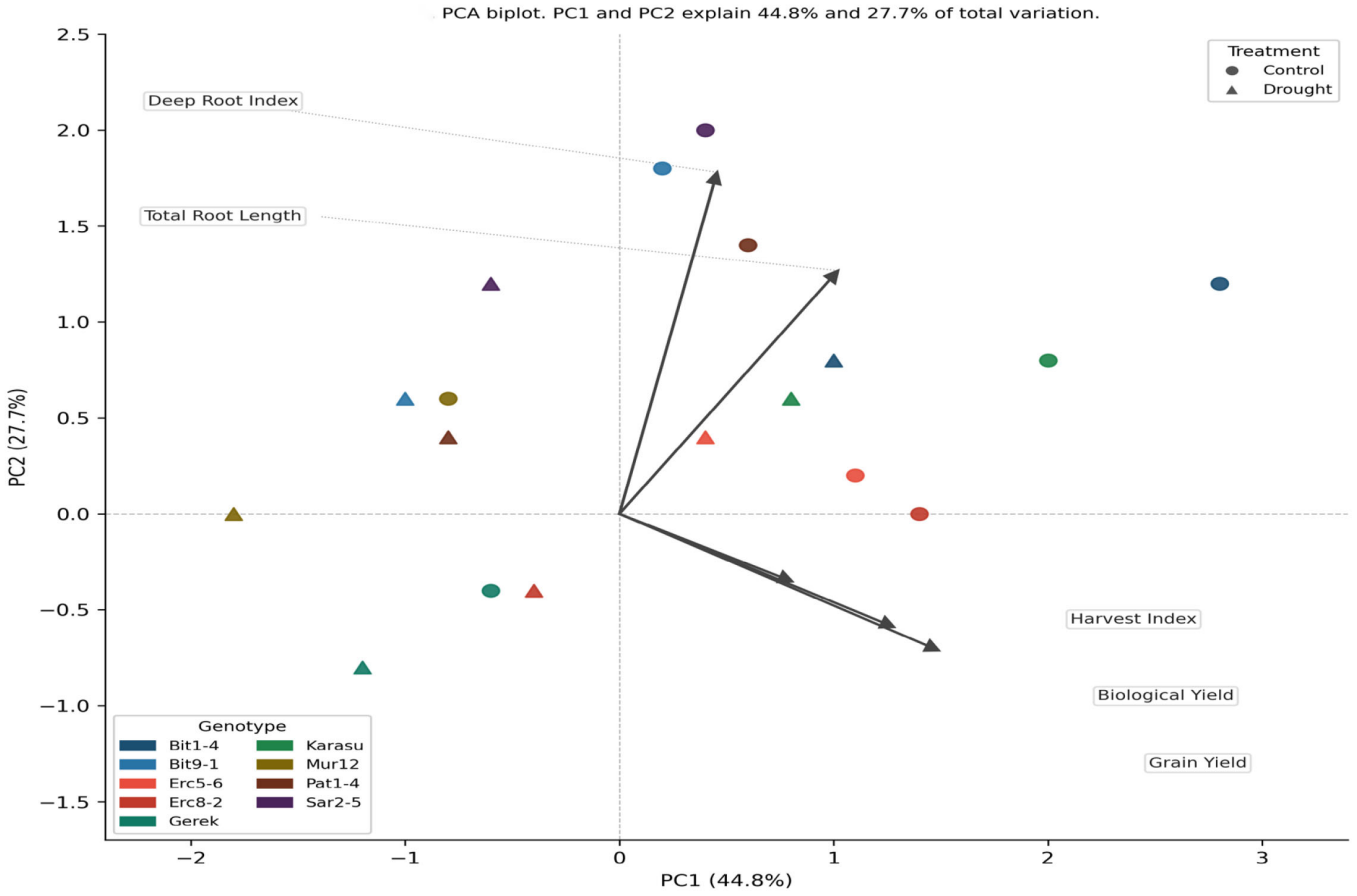
PCA biplot showing relationships among grain yield, biological yield, harvest index, total root length, and deep root index. PC1 and PC2 explained 44.8% and 27.7% of the total variation, respectively. Each point represents the genotype × water-treatment centroid (mean PCA score) for control (circles) or drought (triangles) conditions, averaged across the underlying individual observations; arrows indicate trait loadings.

Principal component analysis (PCA) was performed on five traits grain yield, biological yield, harvest index, total root length, and deep root index using standardized values from all 180 observations. For visual clarity, the PCA biplot (**Figure 3**) displays genotype × water-treatment centroids, calculated by averaging the individual PCA scores within each of the 18 genotype × treatment combinations; all loadings and explained variance, however, were derived from the full set of 180 individual observations. The first two principal components (PC1 and PC2) collectively explained 72.5% of the total variation (PC1: 44.8%; PC2: 27.7%), with PCA loadings presented in **Supplementary Table 8**.

PC1 was primarily associated with yield-related traits, with grain yield (loading = 0.621), biological yield (loading = 0.534), and harvest index (loading = 0.336) showing the highest positive loadings along this axis. Total root length also contributed to PC1 (loading = 0.425), though to a lesser extent than yield traits.

PC2 was dominated by root-related traits, with deep root index showing the highest loading (0.742), followed by total root length (0.528). Yield-related traits showed comparatively low loadings along PC2, indicating limited association between yield performance and root distribution characteristics along this axis.

The PCA biplot (**Figure 3**) showed that grain yield, biological yield, and harvest index were closely grouped and oriented in a similar direction along PC1. In contrast, deep root index and total root length were distributed along PC2, showing limited alignment with yield-related traits. The spatial separation of yield and root traits along different principal component axes is consistent with the weak root-yield correlations reported above.

Overall, the PCA results indicated a clear separation between yield-related and root-related trait groups, with genotypes showing variable associations with each trait cluster across the biplot.

### 3.6 Regression analyses of grain yield on root traits

**Figure 4:**
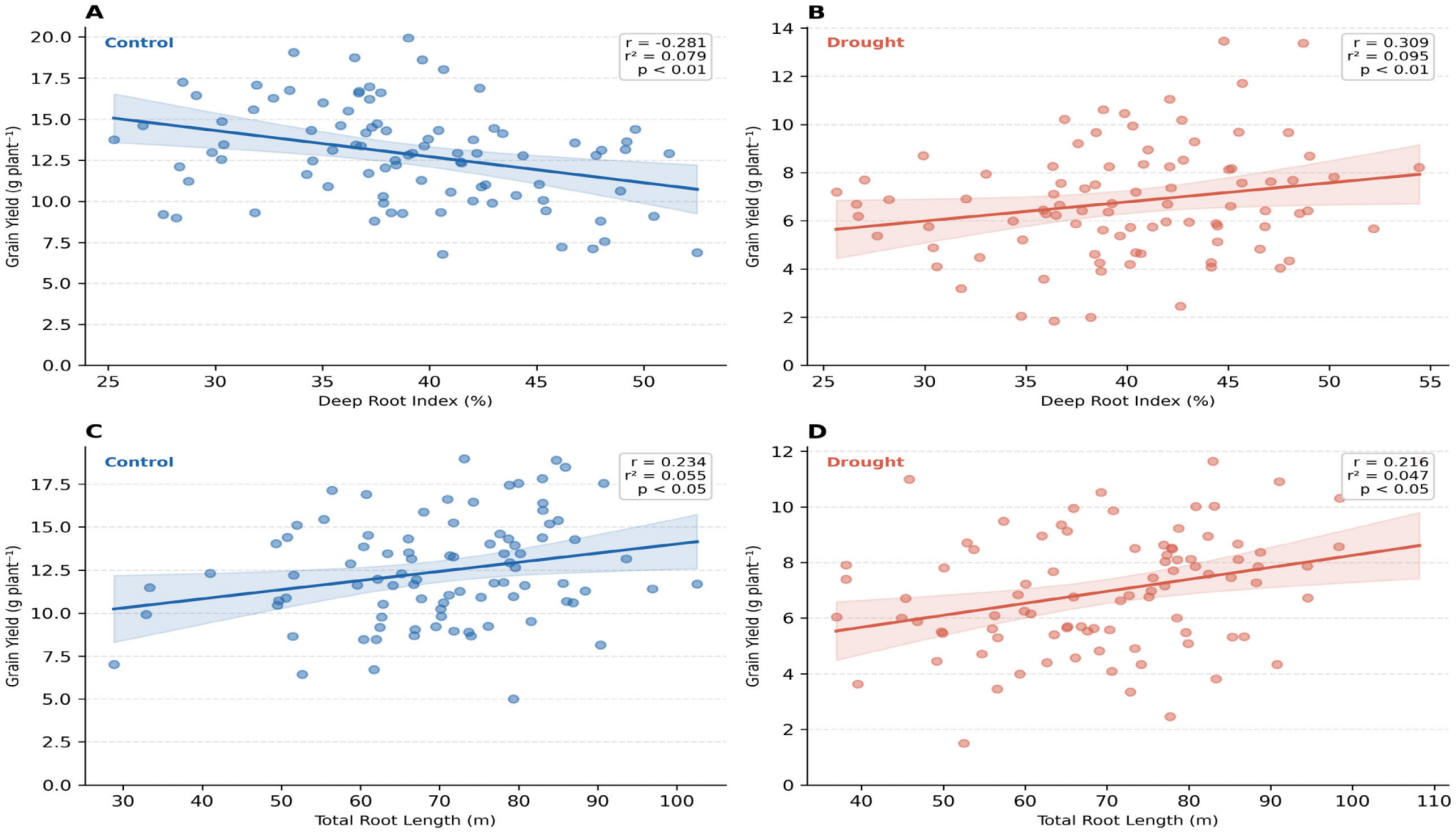
Linear regression of grain yield on **(A, B)** deep root index and **(C, D)** total root length under control and drought conditions. Blue and red points represent control and drought treatments, respectively; shaded areas represent 95% confidence intervals. r: Pearson correlation coefficient; r²: coefficient of determination. *p < 0.05; **p < 0.01; ***p < 0.001; ns, not significant.

Linear regression analyses were performed to further examine the relationships between grain yield and root traits under control and drought conditions separately (**Figure 4**).

Deep root index showed contrasting relationships with grain yield depending on water treatment. Under control conditions, deep root index was negatively associated with grain yield (r = −0.281, r² = 0.079, p = 0.007), whereas under drought conditions the relationship was positive (r = 0.309, r² = 0.095, p = 0.003). This reversal in the direction of association between control and drought conditions suggests that the adaptive value of deep rooting is context-dependent and becomes more pronounced under water-limited conditions.

Total root length showed weak but statistically significant positive associations with grain yield under both control (r = 0.234, r² = 0.055, p = 0.026) and drought conditions (r = 0.216, r² = 0.047, p = 0.041). The low r² values indicate that total root length explains only a small proportion of the variation in grain yield under either condition.

Overall, regression analyses confirmed that deep root index and total root length each explained limited variance in grain yield, with r² values ranging from 0.047 to 0.095. However, the contrasting directional effects of deep root index under control versus drought conditions highlight the importance of water regime in determining the relationship between root distribution and yield performance.

## 4 Discussion

### 4.1 Water availability is the primary driver of yield reduction under drought

The pronounced reduction in grain yield across all genotypes confirms that soil water availability was the dominant factor limiting productivity in this study. The magnitude of the water treatment effect on grain yield (F = 4651.98, p < 0.001) substantially exceeded that of genotype (F = 463.85, p < 0.001), indicating that yield formation was strongly constrained by water deficit regardless of genetic background. Drought stress likely reduced yield through multiple interacting physiological processes, including reduced photosynthetic activity, accelerated leaf senescence, and limited assimilate supply during grain filling, which collectively restrict biomass production and reproductive development (Hajibarat and Saidi, 2022; Nyaupane et al., 2024).

Despite this strong environmental influence, the significant genotype × water interaction (F = 74.11, p < 0.001) demonstrated that genotypes differed considerably in their capacity to maintain yield under drought. Yield retention under drought calculated as the percentage of control yield maintained under stress ranged from 55.2% (Saray 2-5) to 77.9% (Erciş 5-6), indicating substantial genotypic variation in drought response (**Supplementary Table 7**). Similar genotype × treatment interactions for yield-related traits have been reported in controlled greenhouse experiments with wheat, where the G×T interaction F-value for grain weight exceeded 7000 (Nagy et al., 2025), and in field evaluations of diverse wheat panels under contrasting water regimes (Sewore and Abe, 2024). Bitlis 1–4 and Karasu exhibited relatively higher grain yield under drought conditions (11.0 and 9.1 g plant^−1^, respectively), whereas Muradiye 12–2 and Patnos 1–4 showed the greatest yield reductions.

These contrasting responses highlight that yield stability under drought is not solely determined by stress intensity but is also shaped by genotype specific physiological and morphological characteristics. On this basis, genotypes such as Bitlis 1–4 and Erciş 5-6, which combined relatively high yield retention with increased deep root allocation, showed promising characteristics for further evaluation under limited-water or drought-prone field conditions, whereas genotypes such as Erciş 8–2 and Saray 2-5, which achieved their highest absolute yields under well-watered conditions but showed comparatively lower yield retention under drought, may be more suitable candidates for further testing under well-watered or supplementary-irrigation conditions. Karasu, which maintained yield with only moderate root reorganization, may be of interest for intermediate, moderately water-limited environments. These preliminary agronomic implications are based on a single-location greenhouse tube experiment and should be validated through multi-location and multi-year field trials before specific cultivation recommendations are made. Identifying genotypes with consistently high yield retention therefore remains an important objective for breeding programs targeting drought-prone environments (Kamara et al., 2022).

### 4.2 Root system size alone does not explain yield performance under drought

In the present study, root system size showed limited association with grain yield regardless of water treatment. Total root length explained only 5.5% of yield variation under control conditions (r = 0.234, r² = 0.055) and 4.7% under drought (r = 0.216, r² = 0.047), indicating that larger root systems did not consistently translate into higher productivity. Deep root index showed no meaningful association with grain yield overall (r = −0.01, r² < 0.001), further confirming that neither root system size nor total deep root allocation adequately predicted yield performance under the conditions of this study.

**Figure 5:**
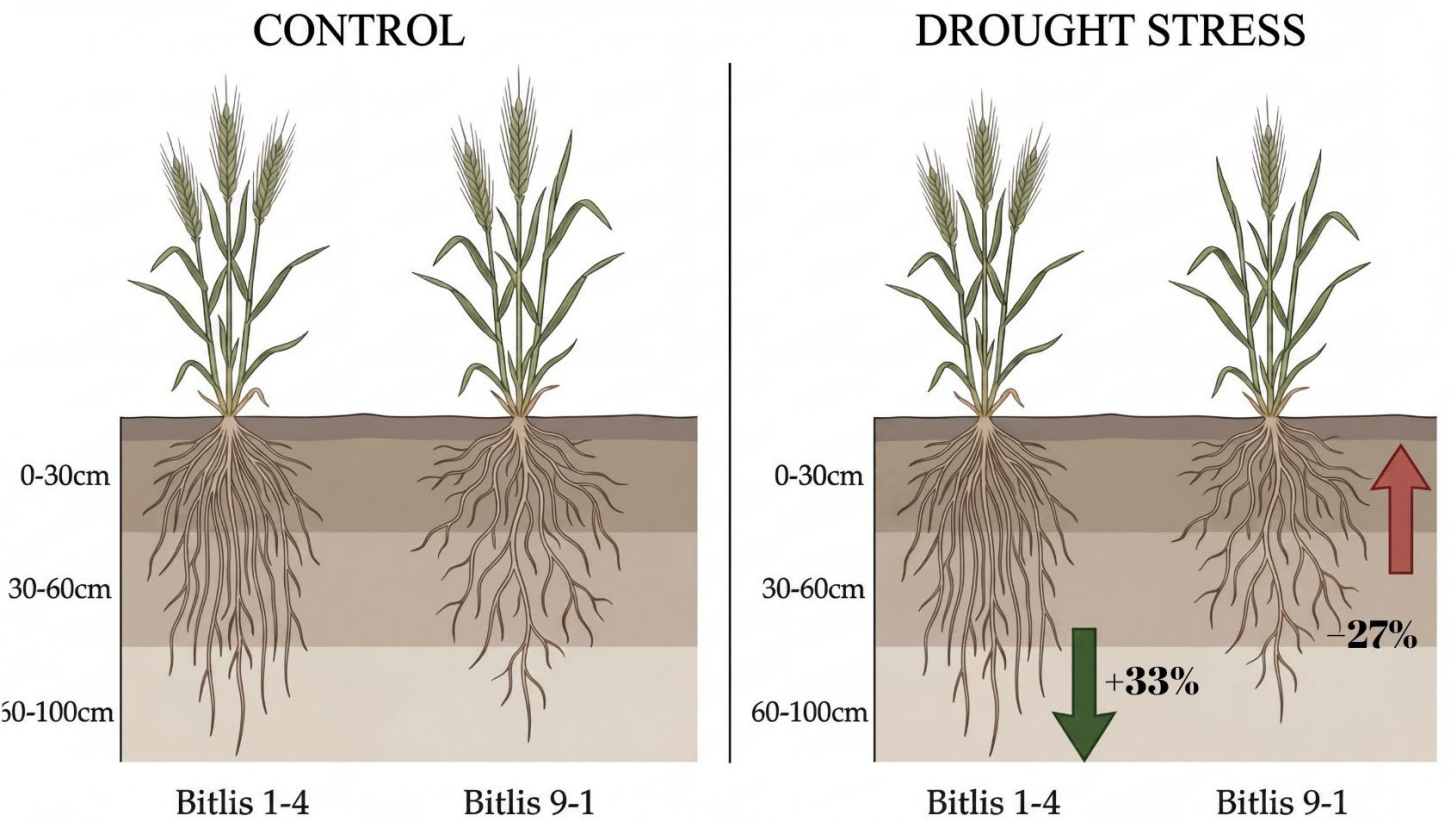
Conceptual illustration of contrasting genotype-specific changes in deep root allocation under drought. Bitlis 1–4 increased its deep root index from 39.8% under control conditions to 53.1% under drought, corresponding to a 33% relative increase. In contrast, Bitlis 9–1 decreased its deep root index from 44.3% to 32.1%, corresponding to a 27% relative reduction. The illustrations are schematic and are not drawn to scale.

The case of Saray 2–5 illustrates this pattern clearly. This genotype exhibited the highest total root length under both control (90.1 m) and drought (82.1 m) conditions, yet its grain yield under drought remained comparatively low (6.9 g plant^−1^). In contrast, Bitlis 1–4 maintained the highest grain yield under drought (11.0 g plant^−1^) despite having a smaller root system (**Figure 5**). This divergence is consistent with the concept of rhizoeconomics, which posits that the construction and maintenance of large root systems imposes a substantial metabolic carbon cost that may reduce the availability of assimilates for reproductive development (Lynch and Ho, 2005; Lynch, 2007). Under drought conditions, genotypes that invest heavily in root biomass may experience a trade-off between belowground resource acquisition and aboveground reproductive allocation, ultimately limiting grain filling capacity and yield formation (Bektas et al., 2023; Fang et al., 2024). These findings are consistent with the view that root system size alone is not a reliable predictor of drought adaptation (Palta and Turner, 2019), and align with recent evidence that root-yield relationships are highly variety-specific in wheat (Durand-Maniclas et al., 2025).

### 4.3 Root distribution across soil depth is a key component of drought adaptation

Soil depth emerged as the dominant source of variation in root traits in this study, with F-values for depth substantially exceeding those for genotype and water treatment (F = 765.63 for root length; F = 2128.39 for surface area). This pronounced depth effect, together with significant genotype × depth, water × depth, and genotype × water × depth interactions, indicates that root distribution along the soil profile is a critical determinant of plant responses to drought. These findings are consistent with the view that vertical root distribution provides important information beyond total root system size for understanding plant adaptation to water deficit (Rathod et al., 2022; Bacher et al., 2023).

Under drought conditions, genotypes differed markedly in their root distribution responses across soil depths. Bitlis 1–4 increased root length in the deepest soil layer (60–100 cm) from 35.79 m to 42.84 m, accompanied by an increase in RLD from 1.14 to 1.36 cm cm^−3^. Similarly, Erciş 5–6 showed a pronounced increase in deep root length from 19.59 m to 34.10 m. The ability to maintain or increase root presence in deeper soil layers may contribute to improved access to soil moisture under drought conditions, particularly when water availability declines in upper layers. Recent evidence supports this interpretation, showing that deep rooting is positively associated with uptake of water from deeper soil layers and is negatively correlated with water stress levels, with this relationship becoming stronger in dry years (Odone et al., 2025).

In contrast, other genotypes showed reduced root length in deeper layers under drought. Bitlis 9–1 decreased from 38.33 m to 17.17 m, and Gerek from 22.92 m to 11.33 m in the 60–100 cm layer. This divergence in depth-dependent root responses highlights that genotypes differ not only in root system size but also in the spatial deployment of roots within the soil profile — a distinction with important implications for drought adaptation and breeding (Makebe et al., 2025; Odone et al., 2025).

The significant three-way interaction between genotype, water treatment, and soil depth (G×W×D) indicates that root distribution patterns are shaped by the combined influence of genetic background and environmental conditions. This underscores the importance of evaluating root systems across soil depth profiles rather than as a single integrated measure, particularly when comparing genotypes for drought adaptation.

### 4.4 Deep rooting is a genotype-dependent trait rather than a direct response to drought

One of the central findings of this study was that deep root index was not significantly affected by water treatment alone (F = 0.70, p > 0.05), whereas genotype (F = 28.03, p < 0.001) and genotype × water interaction (F = 41.19, p < 0.001) were highly significant. This pattern strongly suggests that variation in deep rooting is primarily determined by genetic background rather than being directly induced by drought stress — a finding that has fundamental implications for how root traits are interpreted in the context of drought adaptation.

The contrasting responses observed among genotypes provide clear evidence of this genotype-dependent nature. Bitlis 1–4 increased deep root index from 39.8% to 53.1% under drought (+33.4%), and Erciş 5–6 from 33.0% to 47.6% (+44.2%), indicating an inherent capacity for deeper root allocation under stress. In contrast, Bitlis 9–1 decreased from 44.3% to 32.1% (−27.5%) and Gerek from 41.2% to 28.9% (−29.9%) under the same conditions. These divergent responses under identical experimental conditions indicate that deep rooting reflects inherent differences in root system architecture among genotypes rather than a universally induced response to water deficit (Frantová et al., 2025; Siddiqui et al., 2021).

The genotype-dependent patterns observed in this study are further supported by evidence that root architecture traits in wheat are under strong genetic control, with heritability estimates reaching up to 0.99 for certain root angle and depth related traits (Sallam et al., 2024). The molecular and genetic regulation of root system architecture under drought has been increasingly documented, with specific genes and regulatory pathways identified as determinants of root depth and distribution (Wang et al., 2026; Kaur et al., 2026).

Importantly, regression analysis revealed that the relationship between deep root index and grain yield was not uniform across water treatments. Under drought conditions, deep root index showed a weak but significant positive association with grain yield (r = 0.309, r² = 0.095, p = 0.003), whereas under control conditions the relationship was negative (r = −0.281, r² = 0.079, p = 0.007) (**Figure 4**). This reversal in the direction of association suggests that the adaptive value of deep rooting may be more pronounced under water-limited conditions, though the modest r² values indicate that other factors also contribute substantially to yield variation.

Taken together, these findings indicate that deep rooting capacity is an inherent genotypic characteristic in wheat, and that its expression under drought is modulated by genotype × water interactions rather than being uniformly induced by stress. Identifying genotypes with favorable deep rooting characteristics therefore represents a promising avenue for breeding programs targeting drought-prone environments (Kaur et al., 2026).

### 4.5 Weak association between root traits and yield performance

In the present study, root traits and yield related parameters showed consistently weak associations, indicating a degree of functional separation between belowground and aboveground responses. Grain yield was strongly associated with biological yield and harvest index, whereas relationships with root traits were weak or negligible. Total root length showed a low positive correlation with grain yield (r = 0.34), explaining only 5.5% of yield variation under control and 4.7% under drought conditions. Deep root index showed no meaningful association with grain yield overall (r = −0.01). PCA results reinforced this pattern, with yield-related traits clustering along PC1 (44.8%) and root traits loading primarily on PC2 (27.7%). Similar patterns have been reported in durum wheat (Boudiar et al., 2025) and diverse wheat panels (Kathirvelu et al., 2025).

This pattern was evident in the present study, where genotypes with larger root systems did not consistently achieve higher yields. This functional separation suggests that root system development and yield formation are influenced by partially independent physiological processes. Increased allocation to root growth under drought may enhance the potential for water acquisition, but this does not necessarily translate into improved reproductive performance if aboveground processes such as photosynthetic efficiency, assimilate partitioning, and grain filling capacity are not equally maintained (Bektas et al., 2023; Bursakov et al., 2025). The variety-specific nature of the root-yield relationship further complicates this picture: among ten winter wheat varieties evaluated across contrasting European environments, two showed no significant relationship between root length and grain yield, whereas others showed variety-specific associations with either topsoil or subsoil root traits (Durand-Maniclas et al., 2025).

The case of Saray 2–5 exemplifies this decoupling: this genotype exhibited the largest root system under both control (90.1 m) and drought (82.1 m) conditions yet maintained comparatively low grain yield under drought (6.9 g plant^−1^). Conversely, Bitlis 1–4 combined relatively high yield retention with increased deep root allocation, suggesting that certain genotypes may achieve a more favorable balance between resource acquisition and reproductive allocation.

These findings highlight the importance of adopting an integrated approach that considers both root distribution characteristics and aboveground physiological efficiency when evaluating genotypes for drought adaptation, rather than relying on root traits in isolation.

### 4.6 Genotypes exhibit contrasting drought adaptation strategies

Analysis of drought response indices revealed that the nine wheat genotypes did not respond uniformly to water limitation but instead exhibited three broadly distinct patterns of adaptation (**Supplementary Table 7**).

The first group, represented by Bitlis 1–4 and Erciş 5-6, combined moderate-to-high yield retention (63.2% and 77.9%, respectively) with marked increases in deep root allocation under drought (+33.4% and +44.2%, respectively). This pattern may reflect an integrated strategy in which maintained aboveground productivity is supported by enhanced access to deeper soil moisture reserves (Fang et al., 2024; Odone et al., 2024). The co-occurrence of relatively higher yield and increased deep rooting in these genotypes is consistent with the positive association between deep root index and grain yield observed under drought conditions (r = 0.309, p = 0.003; **Figure 4**).

The second group, represented by Karasu, maintained relatively high yield retention (60.3%) with only moderate changes in root distribution (+13.5%). This pattern suggests that yield stability may be achieved through physiological efficiency — maintaining productivity without substantial reorganization of root architecture — rather than through increased water acquisition from deeper layers (Nakhforoosh et al., 2015; Xiang et al., 2025).

The third group, comprising Gerek and Bitlis 9-1, showed a distinct pattern in which high yield retention (75.2% and 77.3%, respectively — among the highest values observed across all nine genotypes) was decoupled from root distribution responses, as deep root allocation markedly decreased under drought in both genotypes (−29.9% and −27.5%, respectively). This combination indicates that yield stability in these genotypes was not achieved through enhanced deep rooting and instead likely reflects alternative physiological mechanisms, such as conservative water use or osmotic adjustment (Wasson et al., 2012). This pattern reinforces a central conclusion of the present study: deep root allocation under drought is not a prerequisite for yield stability, and genotypes can maintain productivity through root-independent strategies.

These contrasting patterns indicate that drought adaptation in wheat is not governed by a single strategy but involves diverse combinations of root and shoot responses. The identification of genotypes exhibiting favorable trait combinations such as Bitlis 1–4 and Erciş 5-6 — may therefore offer valuable material for breeding programs targeting drought-prone environments (Boudiar et al., 2025; Siddiqui et al., 2023).

### 4.7 Implications for breeding and selection

The results of this study suggest that selection based solely on root system size is insufficient for improving drought adaptation, as neither total root length nor deep root index explained a substantial proportion of yield variation under either water regime. Instead, the combination of genotype-specific root distribution patterns and aboveground physiological efficiency emerges as a more promising framework for evaluating drought adaptation in breeding programs targeting drought-prone environments (Burridge et al., 2022; Shoaib et al., 2022).

The strong genetic control of deep rooting observed in this study reflected in the highly significant genotype and genotype × water interaction effects on deep root index suggests that root distribution characteristics can be targeted through direct selection. Genotypes such as Bitlis 1–4 and Erciş 5-6, which combined relatively higher yield retention with increased deep root allocation under drought, represent promising candidates for breeding programs targeting semi-arid environments. The positive association between deep root index and grain yield under drought conditions (r = 0.309, p = 0.003) further supports the potential value of selecting for deep rooting capacity as part of a multi-trait breeding strategy (Wasson et al., 2014; Ober et al., 2021). In practical terms, these genotypes could serve as direct crossing parents in targeted hybridization programs, and their integration into pre-breeding pipelines would enable the systematic evaluation and transfer of deep rooting capacity into elite genetic backgrounds prior to cultivar development.

Several limitations of this study should be acknowledged. First, the experiment was conducted under controlled greenhouse conditions using PVC tubes filled with a standardized soil mixture, which may not fully replicate the complexity of field environments, including natural soil heterogeneity, microbial communities, and fluctuating water availability. The relationships observed between root distribution and yield performance should therefore be interpreted as reflecting genetic potential under controlled conditions rather than field-level performance. Second, soil moisture monitoring initially relied on TDR probes; however, localized void formation around probe insertion sites compromised measurement accuracy, and a gravimetric approach was subsequently adopted as the primary method. Although gravimetric monitoring ensured reliable moisture control throughout the experiment, this methodological adjustment should be noted when interpreting results. Third, the study included nine genotypes from a single geographic region, which limits the generalizability of the findings to broader wheat germplasm. Fourth, the Pearson correlation, simple linear regression, and PCA analyses (Sections 3.4–3.6) were performed at the level of individual experimental units (e.g., n = 180 for PCA, n = 90 per panel for regression), which include repeated observations of the same genotypes across blocks and growing seasons; these analyses should therefore be regarded as exploratory descriptions of trait associations rather than fully independent statistical tests, and the consistently low r² values reported (≤0.095) were emphasized over p-value significance for this reason. Finally, the absence of field validation means that the adaptive value of the root traits identified here, including the preliminary cultivation implications discussed in Section 4.1, remains to be confirmed under natural, multi-location and multi-year growing conditions.

## 5 Conclusion

The present study demonstrated that deep rooting in bread wheat was more strongly associated with genotype than with the main effect of drought treatment. Although water deficit markedly constrained overall productivity, it did not independently induce consistent changes in deep root allocation across genotypes. Instead, highly significant genotype and genotype × water interaction effects revealed pronounced divergence in root distribution strategies among genotypes. Soil depth emerged as the principal determinant of root trait variability, underscoring that vertical root distribution, rather than total root system size alone, is a critical dimension of drought adaptation.

These findings have direct translational relevance for breeding programs targeting drought-prone environments. The considerable heterogeneity observed among Van Lake Basin landraces in root distribution patterns and yield retention highlights an underexploited reservoir of adaptive diversity. Genotypes such as Bitlis 1-4, which combined superior yield retention with increased deep root allocation under drought, represent valuable donor material for pre-breeding and cultivar development in semi-arid environments. Strategic introgression of such germplasm into elite breeding pools may facilitate the pyramiding of complementary traits, including deeper soil moisture acquisition, physiological resilience, and reproductive stability under drought.

Future research should prioritize multi-environment field validation to determine the extent to which the relationships observed here are maintained under fluctuating pedoclimatic conditions. Integrating root phenotyping with traits related to water use efficiency, assimilate partitioning, phenological plasticity, and genomic architecture will be essential for disentangling the mechanistic basis of drought adaptation in wheat. Ultimately, the results of this study suggest that deep rooting should be regarded not as a universal drought response, but as a genotype-specific adaptive asset whose value must be interpreted within specific environmental and breeding contexts.

## Data Availability

The original contributions presented in the study are included in the article/**Supplementary Material**. Further inquiries can be directed to the corresponding author.
