# Supplementary material for: Deep rooting in bread wheat is more strongly associated with genotypes than drought stress: evidence from Van Lake Basin genotypes
Source: Front Plant Sci. 2026 Jul 22;17:1887215. doi: 10.3389/fpls.2026.1887215 (PMC13437366; doi:10.3389/fpls.2026.1887215)
Supplement: Supplementary file 1 [file Table1.docx]

Supplementary Material

**Supplementary Table S1.** Analysis of variance (ANOVA) for shoot and root traits under different genotypes and water treatments.

**Supplementary Table S1A. ANOVA for shoot traits**

| **Source** | **df** | **Plant Height** | **Spike Length** | **Fertile Spikelets** | **Sterile Spikelets** | **Heading** | **Maturity** | **Grain Yield** | **Biological Yield** | **Harvest Index** | **Tillers** | **Spike Weight** | **Grains per Spike** | **Grain Weight per Spike** | **SPAD** |
| --- | --- | --- | --- | --- | --- | --- | --- | --- | --- | --- | --- | --- | --- | --- | --- |
| Year | 1 | 85.13*** | 50.96*** | 73.19*** | 45.96*** | 134.41*** | 120.85*** | 1742.70*** | 2823.11*** | 68.42*** | 684.78*** | 182.09*** | 0.01ns | 1.87ns | 14.51*** |
| Genotype | 8 | 57.58*** | 263.24*** | 34.03*** | 128.83*** | 6731.33*** | 10983.51*** | 463.85*** | 200.13*** | 184.07*** | 195.40*** | 63.42*** | 442.06*** | 2536.94*** | 378.56*** |
| Water | 1 | 720.17*** | 158.99*** | 311.89*** | 815.63*** | 1.16ns | 8072.63*** | 4651.98*** | 2261.44*** | 467.70*** | 51.54*** | 15.67*** | 287.14*** | 346.29*** | 392.99*** |
| Y×G | 8 | 14.37*** | 2.28* | 13.90*** | 14.99*** | 438.94*** | 868.00*** | 59.46*** | 66.62*** | 38.61*** | 51.18*** | 8.90*** | 2.37* | 5.42*** | 9.92*** |
| Y×W | 1 | 18.60*** | 2.93ns | 7.75** | 4.37* | 0.01ns | 1.12ns | 0.87ns | 18.66*** | 41.61*** | 0.80ns | 0.99ns | 1.41ns | 18.46*** | 4.41* |
| G×W | 8 | 11.10*** | 11.93*** | 6.18*** | 14.52*** | 3.09** | 133.97*** | 74.11*** | 40.24*** | 59.00*** | 16.59*** | 20.82*** | 9.28*** | 76.44*** | 97.50*** |
| Y×G×W | 8 | 3.46** | 3.07** | 5.23*** | 3.68** | 1.88ns | 16.30*** | 4.16*** | 20.34*** | 7.37*** | 2.29* | 15.12*** | 0.27ns | 4.81*** | 4.23*** |
| CV (%) | — | 3.94 | 3.46 | 3.29 | 10.64 | 1.09 | 0.51 | 4.31 | 4.32 | 4.60 | 5.52 | 6.33 | 4.49 | 1.29 | 1.35 |

**Supplementary Table S1B. ANOVA for whole-root traits**

| **Source** | **df** | **Root Length** | **Root Dry Weight** | **Surface Area** | **Volume** | **Deep Root Index** | **Root Fraction (60–100 cm)** | **SRL** | **RTD** | **RLD** | **Root/Shoot** |
| --- | --- | --- | --- | --- | --- | --- | --- | --- | --- | --- | --- |
| Year | 1 | 134.24*** | 74.21*** | 29.96*** | 9.02** | 13.06*** | 13.06*** | 46.84*** | 21.13*** | 134.26*** | 688.88*** |
| Genotype | 8 | 52.40*** | 180.65*** | 51.92*** | 35.28*** | 28.03*** | 28.03*** | 23.71*** | 13.18*** | 52.41*** | 76.59*** |
| Water | 1 | 83.61*** | 456.67*** | 124.91*** | 101.70*** | 0.70ns | 0.70ns | 5.98* | 0.79ns | 83.62*** | 149.56*** |
| Y×G | 8 | 25.42*** | 97.58*** | 21.32*** | 11.80*** | 16.19*** | 16.19*** | 43.81*** | 15.25*** | 25.42*** | 85.19*** |
| Y×W | 1 | 12.20*** | 140.34*** | 21.89*** | 19.90*** | 0.06ns | 0.06ns | 13.03*** | 0.04ns | 12.19*** | 193.99*** |
| G×W | 8 | 15.52*** | 44.00*** | 8.08*** | 4.74*** | 41.19*** | 41.19*** | 15.28*** | 6.94*** | 15.52*** | 31.95*** |
| Y×G×W | 8 | 5.67*** | 17.48*** | 3.83*** | 3.18** | 20.68*** | 20.68*** | 12.10*** | 5.03*** | 5.67*** | 13.53*** |
| CV (%) | — | 10.79 | 6.51 | 10.75 | 12.96 | 8.62 | 8.62 | 11.35 | 20.32 | 10.60 | 7.01 |

ns: not significant; * p < 0.05; ** p < 0.01; *** p < 0.001. Y: Year; G: Genotype; W: Water treatment.

**Supplementary Table S2.** Analysis of variance (ANOVA) for depth-dependent root traits.

| **Source** | **df** | **Root Length** | **Dry Weight** | **Diameter** | **Surface Area** | **Volume** | **SRL** | **RTD** | **RLD** |
| --- | --- | --- | --- | --- | --- | --- | --- | --- | --- |
| Year | 1 | 210.485*** | 86.513*** | 164.542*** | 39.751*** | 9.959** | 233.475*** | 9.740** | 221.958*** |
| Genotype | 8 | 82.168*** | 210.601*** | 4.900*** | 68.897*** | 38.970*** | 45.837*** | 10.797*** | 83.965*** |
| Water | 1 | 131.100*** | 532.374*** | 6.892** | 165.742*** | 112.346*** | 20.425*** | 3.702ns | 148.741*** |
| Depth | 2 | 765.630*** | 13729.887*** | 4810.727*** | 2128.385*** | 3455.463*** | 3826.608*** | 14.862*** | 648.861*** |
| G×W | 8 | 24.336*** | 51.292*** | 1.994* | 10.715*** | 5.232*** | 34.067*** | 6.601*** | 21.847*** |
| G×D | 16 | 17.597*** | 60.726*** | 1.750* | 12.240*** | 12.016*** | 23.878*** | 2.742*** | 15.029*** |
| W×D | 2 | 17.420*** | 169.726*** | 0.585ns | 39.707*** | 46.321*** | 16.918*** | 0.359ns | 24.749*** |
| G×W×D | 16 | 21.819*** | 16.783*** | 0.884ns | 12.245*** | 7.204*** | 30.049*** | 2.677*** | 19.871*** |
| CV (%) | — | 14.9 | 8.7 | 9.3 | 16.1 | 21.4 | 17.4 | 36.3 | 14.1 |

ns: not significant; * p < 0.05; ** p < 0.01; *** p < 0.001. G: Genotype; W: Water treatment; D: Soil depth.

**Supplementary Table S3.** Depth-dependent root traits (mean ± SE) across genotypes, water treatments, and soil depths.

| **Year** | **Genotype** | **Water** | **Depth (cm)** | **Length (m)** | **Dry Weight (g)** | **Diameter (mm)** | **Surface Area (cm²)** | **Volume (cm³)** | **SRL (m g⁻¹)** | **RLD (m cm⁻³)** | **RTD (g cm⁻³)** |
| --- | --- | --- | --- | --- | --- | --- | --- | --- | --- | --- | --- |
| 2021 | Bitlis 1-4 | Control | 0–30 | 27.58 ± 1.57 | 1.14 ± 0.02 | 0.50 ± 0.01 | 438.26 ± 17.90 | 5.64 ± 0.23 | 24.26 ± 8.18 | 1.17 ± 0.06 | 0.24 ± 0.03 |
|  |  |  | 30–60 | 21.27 ± 1.57 | 0.33 ± 0.02 | 0.33 ± 0.01 | 219.36 ± 17.90 | 1.82 ± 0.23 | 64.93 ± 8.18 | 0.90 ± 0.06 | 0.20 ± 0.03 |
|  |  |  | 60–100 | 32.19 ± 1.57 | 0.30 ± 0.02 | 0.21 ± 0.01 | 210.34 ± 17.90 | 1.09 ± 0.23 | 109.37 ± 8.18 | 1.03 ± 0.06 | 0.27 ± 0.03 |
|  |  | Drought | 0–30 | 20.85 ± 1.57 | 0.92 ± 0.02 | 0.55 ± 0.01 | 358.61 ± 17.90 | 4.91 ± 0.23 | 22.62 ± 8.18 | 0.89 ± 0.06 | 0.19 ± 0.03 |
|  |  |  | 30–60 | 15.11 ± 1.57 | 0.26 ± 0.02 | 0.33 ± 0.01 | 156.50 ± 17.90 | 1.31 ± 0.23 | 59.33 ± 8.18 | 0.64 ± 0.06 | 0.21 ± 0.03 |
|  |  |  | 60–100 | 46.08 ± 1.57 | 0.22 ± 0.02 | 0.19 ± 0.01 | 279.15 ± 17.90 | 1.38 ± 0.23 | 205.89 ± 8.18 | 1.47 ± 0.06 | 0.20 ± 0.03 |
|  | Bitlis 9-1 | Control | 0–30 | 24.74 ± 1.57 | 0.66 ± 0.02 | 0.51 ± 0.01 | 393.44 ± 17.90 | 5.09 ± 0.23 | 37.71 ± 8.18 | 1.05 ± 0.06 | 0.14 ± 0.03 |
|  |  |  | 30–60 | 16.93 ± 1.57 | 0.28 ± 0.02 | 0.30 ± 0.01 | 163.59 ± 17.90 | 1.28 ± 0.23 | 60.46 ± 8.18 | 0.72 ± 0.06 | 0.25 ± 0.03 |
|  |  |  | 60–100 | 34.21 ± 1.57 | 0.16 ± 0.02 | 0.19 ± 0.01 | 203.61 ± 17.90 | 0.99 ± 0.23 | 213.90 ± 8.18 | 1.09 ± 0.06 | 0.19 ± 0.03 |
|  |  | Drought | 0–30 | 25.90 ± 1.57 | 0.57 ± 0.02 | 0.53 ± 0.01 | 434.56 ± 17.90 | 5.80 ± 0.23 | 45.13 ± 8.18 | 1.10 ± 0.06 | 0.10 ± 0.03 |
|  |  |  | 30–60 | 11.42 ± 1.57 | 0.17 ± 0.02 | 0.32 ± 0.01 | 116.08 ± 17.90 | 0.95 ± 0.23 | 65.53 ± 8.18 | 0.49 ± 0.06 | 0.20 ± 0.03 |
|  |  |  | 60–100 | 11.93 ± 1.57 | 0.10 ± 0.02 | 0.19 ± 0.01 | 72.63 ± 17.90 | 0.36 ± 0.23 | 114.95 ± 8.18 | 0.38 ± 0.06 | 0.31 ± 0.03 |
|  | Erciş 5-6 | Control | 0–30 | 22.67 ± 1.57 | 0.61 ± 0.02 | 0.54 ± 0.01 | 385.04 ± 17.90 | 5.20 ± 0.23 | 36.98 ± 8.18 | 0.96 ± 0.06 | 0.12 ± 0.03 |
|  |  |  | 30–60 | 12.77 ± 1.57 | 0.18 ± 0.02 | 0.34 ± 0.01 | 136.86 ± 17.90 | 1.17 ± 0.23 | 71.73 ± 8.18 | 0.54 ± 0.06 | 0.15 ± 0.03 |
|  |  |  | 60–100 | 14.28 ± 1.57 | 0.12 ± 0.02 | 0.21 ± 0.01 | 94.61 ± 17.90 | 0.50 ± 0.23 | 122.61 ± 8.18 | 0.45 ± 0.06 | 0.24 ± 0.03 |
|  |  | Drought | 0–30 | 15.33 ± 1.57 | 0.62 ± 0.02 | 0.50 ± 0.01 | 241.60 ± 17.90 | 3.10 ± 0.23 | 24.62 ± 8.18 | 0.65 ± 0.06 | 0.23 ± 0.03 |
|  |  |  | 30–60 | 13.37 ± 1.57 | 0.18 ± 0.02 | 0.34 ± 0.01 | 144.33 ± 17.90 | 1.24 ± 0.23 | 74.42 ± 8.18 | 0.57 ± 0.06 | 0.15 ± 0.03 |
|  |  |  | 60–100 | 21.20 ± 1.57 | 0.12 ± 0.02 | 0.18 ± 0.01 | 121.86 ± 17.90 | 0.59 ± 0.23 | 184.71 ± 8.18 | 0.68 ± 0.06 | 0.27 ± 0.03 |
|  | Erciş 8-2 | Control | 0–30 | 25.79 ± 1.57 | 0.90 ± 0.02 | 0.51 ± 0.01 | 405.87 ± 17.90 | 5.20 ± 0.23 | 28.53 ± 8.18 | 1.09 ± 0.06 | 0.19 ± 0.03 |
|  |  |  | 30–60 | 15.81 ± 1.57 | 0.26 ± 0.02 | 0.33 ± 0.01 | 162.68 ± 17.90 | 1.35 ± 0.23 | 60.93 ± 8.18 | 0.67 ± 0.06 | 0.21 ± 0.03 |
|  |  |  | 60–100 | 32.64 ± 1.57 | 0.22 ± 0.02 | 0.21 ± 0.01 | 216.45 ± 17.90 | 1.14 ± 0.23 | 145.92 ± 8.18 | 1.04 ± 0.06 | 0.20 ± 0.03 |
|  |  | Drought | 0–30 | 19.55 ± 1.57 | 0.82 ± 0.02 | 0.55 ± 0.01 | 335.01 ± 17.90 | 4.57 ± 0.23 | 23.82 ± 8.18 | 0.83 ± 0.06 | 0.18 ± 0.03 |
|  |  |  | 30–60 | 13.28 ± 1.57 | 0.32 ± 0.02 | 0.33 ± 0.01 | 129.92 ± 17.90 | 1.10 ± 0.23 | 41.00 ± 8.18 | 0.56 ± 0.06 | 0.34 ± 0.03 |
|  |  |  | 60–100 | 20.69 ± 1.57 | 0.19 ± 0.02 | 0.20 ± 0.01 | 131.11 ± 17.90 | 0.68 ± 0.23 | 110.20 ± 8.18 | 0.66 ± 0.06 | 0.45 ± 0.03 |
|  | Gerek-79 | Control | 0–30 | 17.95 ± 1.57 | 0.55 ± 0.02 | 0.55 ± 0.01 | 307.57 ± 17.90 | 4.19 ± 0.23 | 32.94 ± 8.18 | 0.76 ± 0.06 | 0.13 ± 0.03 |
|  |  |  | 30–60 | 10.49 ± 1.57 | 0.17 ± 0.02 | 0.35 ± 0.01 | 113.52 ± 17.90 | 0.98 ± 0.23 | 63.15 ± 8.18 | 0.45 ± 0.06 | 0.17 ± 0.03 |
|  |  |  | 60–100 | 17.39 ± 1.57 | 0.11 ± 0.02 | 0.20 ± 0.01 | 107.99 ± 17.90 | 0.55 ± 0.23 | 160.72 ± 8.18 | 0.55 ± 0.06 | 0.23 ± 0.03 |
|  |  | Drought | 0–30 | 17.20 ± 1.57 | 0.51 ± 0.02 | 0.51 ± 0.01 | 273.39 ± 17.90 | 3.54 ± 0.23 | 33.94 ± 8.18 | 0.73 ± 0.06 | 0.16 ± 0.03 |
|  |  |  | 30–60 | 8.82 ± 1.57 | 0.16 ± 0.02 | 0.35 ± 0.01 | 96.57 ± 17.90 | 0.84 ± 0.23 | 54.45 ± 8.18 | 0.37 ± 0.06 | 0.19 ± 0.03 |
|  |  |  | 60–100 | 13.48 ± 1.57 | 0.10 ± 0.02 | 0.22 ± 0.01 | 92.12 ± 17.90 | 0.50 ± 0.23 | 131.38 ± 8.18 | 0.43 ± 0.06 | 0.21 ± 0.03 |
|  | Karasu | Control | 0–30 | 21.13 ± 1.57 | 0.58 ± 0.02 | 0.54 ± 0.01 | 360.20 ± 17.90 | 4.89 ± 0.23 | 36.54 ± 8.18 | 0.90 ± 0.06 | 0.12 ± 0.03 |
|  |  |  | 30–60 | 10.04 ± 1.57 | 0.16 ± 0.02 | 0.35 ± 0.01 | 109.08 ± 17.90 | 0.94 ± 0.23 | 61.92 ± 8.18 | 0.43 ± 0.06 | 0.17 ± 0.03 |
|  |  |  | 60–100 | 15.01 ± 1.57 | 0.11 ± 0.02 | 0.21 ± 0.01 | 99.95 ± 17.90 | 0.53 ± 0.23 | 135.40 ± 8.18 | 0.48 ± 0.06 | 0.21 ± 0.03 |
|  |  | Drought | 0–30 | 27.78 ± 1.57 | 0.59 ± 0.02 | 0.53 ± 0.01 | 465.82 ± 17.90 | 6.22 ± 0.23 | 46.81 ± 8.18 | 1.18 ± 0.06 | 0.10 ± 0.03 |
|  |  |  | 30–60 | 9.16 ± 1.57 | 0.16 ± 0.02 | 0.32 ± 0.01 | 92.22 ± 17.90 | 0.75 ± 0.23 | 56.95 ± 8.18 | 0.39 ± 0.06 | 0.23 ± 0.03 |
|  |  |  | 60–100 | 15.93 ± 1.57 | 0.11 ± 0.02 | 0.21 ± 0.01 | 103.02 ± 17.90 | 0.53 ± 0.23 | 152.07 ± 8.18 | 0.51 ± 0.06 | 0.20 ± 0.03 |
|  | Muradiye 12-2 | Control | 0–30 | 25.48 ± 1.57 | 0.58 ± 0.02 | 0.54 ± 0.01 | 433.84 ± 17.90 | 5.88 ± 0.23 | 44.12 ± 8.18 | 1.08 ± 0.06 | 0.10 ± 0.03 |
|  |  |  | 30–60 | 11.72 ± 1.57 | 0.17 ± 0.02 | 0.33 ± 0.01 | 119.50 ± 17.90 | 0.98 ± 0.23 | 69.05 ± 8.18 | 0.50 ± 0.06 | 0.18 ± 0.03 |
|  |  |  | 60–100 | 16.70 ± 1.57 | 0.12 ± 0.02 | 0.21 ± 0.01 | 110.33 ± 17.90 | 0.58 ± 0.23 | 142.21 ± 8.18 | 0.53 ± 0.06 | 0.20 ± 0.03 |
|  |  | Drought | 0–30 | 24.48 ± 1.57 | 0.58 ± 0.02 | 0.54 ± 0.01 | 416.83 ± 17.90 | 5.65 ± 0.23 | 42.27 ± 8.18 | 1.04 ± 0.06 | 0.10 ± 0.03 |
|  |  |  | 30–60 | 10.36 ± 1.57 | 0.16 ± 0.02 | 0.33 ± 0.01 | 105.90 ± 17.90 | 0.87 ± 0.23 | 63.80 ± 8.18 | 0.44 ± 0.06 | 0.20 ± 0.03 |
|  |  |  | 60–100 | 20.93 ± 1.57 | 0.12 ± 0.02 | 0.19 ± 0.01 | 123.70 ± 17.90 | 0.60 ± 0.23 | 182.37 ± 8.18 | 0.67 ± 0.06 | 0.23 ± 0.03 |
|  | Patnos 1-4 | Control | 0–30 | 34.17 ± 1.57 | 0.58 ± 0.02 | 0.54 ± 0.01 | 576.14 ± 17.90 | 7.73 ± 0.23 | 59.45 ± 8.18 | 1.45 ± 0.06 | 0.08 ± 0.03 |
|  |  |  | 30–60 | 21.81 ± 1.57 | 0.18 ± 0.02 | 0.35 ± 0.01 | 236.54 ± 17.90 | 2.04 ± 0.23 | 123.00 ± 8.18 | 0.93 ± 0.06 | 0.09 ± 0.03 |
|  |  |  | 60–100 | 42.06 ± 1.57 | 0.11 ± 0.02 | 0.21 ± 0.01 | 274.75 ± 17.90 | 1.43 ± 0.23 | 371.85 ± 8.18 | 1.34 ± 0.06 | 0.08 ± 0.03 |
|  |  | Drought | 0–30 | 27.55 ± 1.57 | 0.57 ± 0.02 | 0.50 ± 0.01 | 435.14 ± 17.90 | 5.58 ± 0.23 | 48.80 ± 8.18 | 1.17 ± 0.06 | 0.12 ± 0.03 |
|  |  |  | 30–60 | 20.08 ± 1.57 | 0.18 ± 0.02 | 0.33 ± 0.01 | 207.19 ± 17.90 | 1.72 ± 0.23 | 112.47 ± 8.18 | 0.85 ± 0.06 | 0.12 ± 0.03 |
|  |  |  | 60–100 | 38.73 ± 1.57 | 0.12 ± 0.02 | 0.21 ± 0.01 | 257.42 ± 17.90 | 1.36 ± 0.23 | 316.07 ± 8.18 | 1.23 ± 0.06 | 0.10 ± 0.03 |
|  | Saray 2-5 | Control | 0–30 | 28.47 ± 1.57 | 0.81 ± 0.02 | 0.54 ± 0.01 | 485.94 ± 17.90 | 6.60 ± 0.23 | 35.05 ± 8.18 | 1.21 ± 0.06 | 0.12 ± 0.03 |
|  |  |  | 30–60 | 15.16 ± 1.57 | 0.23 ± 0.02 | 0.33 ± 0.01 | 156.24 ± 17.90 | 1.30 ± 0.23 | 65.36 ± 8.18 | 0.64 ± 0.06 | 0.20 ± 0.03 |
|  |  |  | 60–100 | 34.05 ± 1.57 | 0.24 ± 0.02 | 0.21 ± 0.01 | 227.10 ± 17.90 | 1.21 ± 0.23 | 144.18 ± 8.18 | 1.08 ± 0.06 | 0.21 ± 0.03 |
|  |  | Drought | 0–30 | 28.04 ± 1.57 | 0.68 ± 0.02 | 0.55 ± 0.01 | 480.56 ± 17.90 | 6.56 ± 0.23 | 41.26 ± 8.18 | 1.19 ± 0.06 | 0.10 ± 0.03 |
|  |  |  | 30–60 | 16.26 ± 1.57 | 0.22 ± 0.02 | 0.33 ± 0.01 | 168.46 ± 17.90 | 1.41 ± 0.23 | 73.29 ± 8.18 | 0.69 ± 0.06 | 0.17 ± 0.03 |
|  |  |  | 60–100 | 31.39 ± 1.57 | 0.18 ± 0.02 | 0.22 ± 0.01 | 212.95 ± 17.90 | 1.15 ± 0.23 | 173.21 ± 8.18 | 1.00 ± 0.06 | 0.16 ± 0.03 |
| 2022 | Bitlis 1-4 | Control | 0–30 | 39.01 ± 1.57 | 0.96 ± 0.02 | 0.45 ± 0.01 | 551.34 ± 17.90 | 6.20 ± 0.23 | 41.22 ± 8.18 | 1.66 ± 0.06 | 0.16 ± 0.03 |
|  |  |  | 30–60 | 20.66 ± 1.57 | 0.15 ± 0.02 | 0.33 ± 0.01 | 210.10 ± 17.90 | 1.72 ± 0.23 | 135.54 ± 8.18 | 0.88 ± 0.06 | 0.09 ± 0.03 |
|  |  |  | 60–100 | 39.39 ± 1.57 | 0.17 ± 0.02 | 0.22 ± 0.01 | 266.90 ± 17.90 | 1.44 ± 0.23 | 237.52 ± 8.18 | 1.25 ± 0.06 | 0.12 ± 0.03 |
|  |  | Drought | 0–30 | 24.34 ± 1.57 | 0.55 ± 0.02 | 0.44 ± 0.01 | 338.63 ± 17.90 | 3.75 ± 0.23 | 44.55 ± 8.18 | 1.03 ± 0.06 | 0.15 ± 0.03 |
|  |  |  | 30–60 | 15.05 ± 1.57 | 0.14 ± 0.02 | 0.32 ± 0.01 | 152.67 ± 17.90 | 1.23 ± 0.23 | 108.10 ± 8.18 | 0.64 ± 0.06 | 0.12 ± 0.03 |
|  |  |  | 60–100 | 39.59 ± 1.57 | 0.11 ± 0.02 | 0.21 ± 0.01 | 254.16 ± 17.90 | 1.31 ± 0.23 | 366.97 ± 8.18 | 1.26 ± 0.06 | 0.09 ± 0.03 |
|  | Bitlis 9-1 | Control | 0–30 | 35.37 ± 1.57 | 1.07 ± 0.02 | 0.45 ± 0.01 | 504.98 ± 17.90 | 5.74 ± 0.23 | 33.29 ± 8.18 | 1.50 ± 0.06 | 0.19 ± 0.03 |
|  |  |  | 30–60 | 20.06 ± 1.57 | 0.28 ± 0.02 | 0.32 ± 0.01 | 203.25 ± 17.90 | 1.64 ± 0.23 | 73.15 ± 8.18 | 0.85 ± 0.06 | 0.17 ± 0.03 |
|  |  |  | 60–100 | 42.44 ± 1.57 | 0.18 ± 0.02 | 0.20 ± 0.01 | 268.76 ± 17.90 | 1.36 ± 0.23 | 244.91 ± 8.18 | 1.35 ± 0.06 | 0.14 ± 0.03 |
|  |  | Drought | 0–30 | 19.29 ± 1.57 | 0.45 ± 0.02 | 0.45 ± 0.01 | 273.61 ± 17.90 | 3.09 ± 0.23 | 42.86 ± 8.18 | 0.82 ± 0.06 | 0.15 ± 0.03 |
|  |  |  | 30–60 | 14.38 ± 1.57 | 0.14 ± 0.02 | 0.32 ± 0.01 | 144.33 ± 17.90 | 1.16 ± 0.23 | 101.87 ± 8.18 | 0.61 ± 0.06 | 0.12 ± 0.03 |
|  |  |  | 60–100 | 22.40 ± 1.57 | 0.09 ± 0.02 | 0.21 ± 0.01 | 145.70 ± 17.90 | 0.75 ± 0.23 | 248.93 ± 8.18 | 0.71 ± 0.06 | 0.12 ± 0.03 |
|  | Erciş 5-6 | Control | 0–30 | 26.82 ± 1.57 | 0.84 ± 0.02 | 0.49 ± 0.01 | 415.17 ± 17.90 | 5.12 ± 0.23 | 32.12 ± 8.18 | 1.14 ± 0.06 | 0.16 ± 0.03 |
|  |  |  | 30–60 | 15.04 ± 1.57 | 0.23 ± 0.02 | 0.35 ± 0.01 | 166.29 ± 17.90 | 1.46 ± 0.23 | 64.76 ± 8.18 | 0.64 ± 0.06 | 0.16 ± 0.03 |
|  |  |  | 60–100 | 24.91 ± 1.57 | 0.24 ± 0.02 | 0.22 ± 0.01 | 169.11 ± 17.90 | 0.91 ± 0.23 | 103.42 ± 8.18 | 0.79 ± 0.06 | 0.27 ± 0.03 |
|  |  | Drought | 0–30 | 16.46 ± 1.57 | 0.55 ± 0.02 | 0.50 ± 0.01 | 258.34 ± 17.90 | 3.23 ± 0.23 | 30.37 ± 8.18 | 0.70 ± 0.06 | 0.18 ± 0.03 |
|  |  |  | 30–60 | 25.37 ± 1.57 | 0.24 ± 0.02 | 0.35 ± 0.01 | 280.37 ± 17.90 | 2.47 ± 0.23 | 106.90 ± 8.18 | 1.08 ± 0.06 | 0.10 ± 0.03 |
|  |  |  | 60–100 | 47.01 ± 1.57 | 0.16 ± 0.02 | 0.19 ± 0.01 | 272.80 ± 17.90 | 1.26 ± 0.23 | 296.59 ± 8.18 | 1.50 ± 0.06 | 0.13 ± 0.03 |
|  | Erciş 8-2 | Control | 0–30 | 37.63 ± 1.57 | 0.82 ± 0.02 | 0.43 ± 0.01 | 512.69 ± 17.90 | 5.56 ± 0.23 | 45.74 ± 8.18 | 1.60 ± 0.06 | 0.15 ± 0.03 |
|  |  |  | 30–60 | 13.56 ± 1.57 | 0.27 ± 0.02 | 0.33 ± 0.01 | 138.45 ± 17.90 | 1.13 ± 0.23 | 50.01 ± 8.18 | 0.58 ± 0.06 | 0.25 ± 0.03 |
|  |  |  | 60–100 | 32.77 ± 1.57 | 0.17 ± 0.02 | 0.21 ± 0.01 | 214.75 ± 17.90 | 1.12 ± 0.23 | 191.14 ± 8.18 | 1.04 ± 0.06 | 0.15 ± 0.03 |
|  |  | Drought | 0–30 | 20.21 ± 1.57 | 0.74 ± 0.02 | 0.42 ± 0.01 | 265.70 ± 17.90 | 2.78 ± 0.23 | 27.48 ± 8.18 | 0.86 ± 0.06 | 0.27 ± 0.03 |
|  |  |  | 30–60 | 19.86 ± 1.57 | 0.30 ± 0.02 | 0.31 ± 0.01 | 192.80 ± 17.90 | 1.49 ± 0.23 | 66.26 ± 8.18 | 0.84 ± 0.06 | 0.21 ± 0.03 |
|  |  |  | 60–100 | 28.76 ± 1.57 | 0.13 ± 0.02 | 0.18 ± 0.01 | 166.09 ± 17.90 | 0.76 ± 0.23 | 211.07 ± 8.18 | 0.92 ± 0.06 | 0.18 ± 0.03 |
|  | Gerek-79 | Control | 0–30 | 25.92 ± 1.57 | 0.45 ± 0.02 | 0.43 ± 0.01 | 348.37 ± 17.90 | 3.73 ± 0.23 | 57.60 ± 8.18 | 1.10 ± 0.06 | 0.12 ± 0.03 |
|  |  |  | 30–60 | 9.55 ± 1.57 | 0.14 ± 0.02 | 0.30 ± 0.01 | 91.20 ± 17.90 | 0.69 ± 0.23 | 70.74 ± 8.18 | 0.41 ± 0.06 | 0.21 ± 0.03 |
|  |  |  | 60–100 | 28.45 ± 1.57 | 0.09 ± 0.02 | 0.20 ± 0.01 | 177.44 ± 17.90 | 0.88 ± 0.23 | 312.47 ± 8.18 | 0.91 ± 0.06 | 0.11 ± 0.03 |
|  |  | Drought | 0–30 | 22.34 ± 1.57 | 0.45 ± 0.02 | 0.42 ± 0.01 | 295.14 ± 17.90 | 3.11 ± 0.23 | 49.64 ± 8.18 | 0.95 ± 0.06 | 0.15 ± 0.03 |
|  |  |  | 30–60 | 7.21 ± 1.57 | 0.14 ± 0.02 | 0.30 ± 0.01 | 68.31 ± 17.90 | 0.52 ± 0.23 | 53.41 ± 8.18 | 0.31 ± 0.06 | 0.26 ± 0.03 |
|  |  |  | 60–100 | 9.18 ± 1.57 | 0.09 ± 0.02 | 0.18 ± 0.01 | 51.30 ± 17.90 | 0.23 ± 0.23 | 102.00 ± 8.18 | 0.29 ± 0.06 | 0.40 ± 0.03 |
|  | Karasu | Control | 0–30 | 32.84 ± 1.57 | 0.79 ± 0.02 | 0.42 ± 0.01 | 436.76 ± 17.90 | 4.63 ± 0.23 | 41.64 ± 8.18 | 1.39 ± 0.06 | 0.17 ± 0.03 |
|  |  |  | 30–60 | 16.96 ± 1.57 | 0.17 ± 0.02 | 0.30 ± 0.01 | 159.63 ± 17.90 | 1.20 ± 0.23 | 99.71 ± 8.18 | 0.72 ± 0.06 | 0.14 ± 0.03 |
|  |  |  | 60–100 | 20.20 ± 1.57 | 0.09 ± 0.02 | 0.18 ± 0.01 | 114.71 ± 17.90 | 0.52 ± 0.23 | 224.44 ± 8.18 | 0.64 ± 0.06 | 0.17 ± 0.03 |
|  |  | Drought | 0–30 | 30.81 ± 1.57 | 0.93 ± 0.02 | 0.42 ± 0.01 | 403.99 ± 17.90 | 4.22 ± 0.23 | 33.12 ± 8.18 | 1.31 ± 0.06 | 0.23 ± 0.03 |
|  |  |  | 30–60 | 15.45 ± 1.57 | 0.17 ± 0.02 | 0.29 ± 0.01 | 140.05 ± 17.90 | 1.01 ± 0.23 | 89.38 ± 8.18 | 0.66 ± 0.06 | 0.18 ± 0.03 |
|  |  |  | 60–100 | 30.72 ± 1.57 | 0.09 ± 0.02 | 0.18 ± 0.01 | 171.58 ± 17.90 | 0.76 ± 0.23 | 321.93 ± 8.18 | 0.98 ± 0.06 | 0.13 ± 0.03 |
|  | Muradiye 12-2 | Control | 0–30 | 30.82 ± 1.57 | 0.99 ± 0.02 | 0.51 ± 0.01 | 496.51 ± 17.90 | 6.37 ± 0.23 | 31.31 ± 8.18 | 1.31 ± 0.06 | 0.16 ± 0.03 |
|  |  |  | 30–60 | 15.39 ± 1.57 | 0.17 ± 0.02 | 0.35 ± 0.01 | 167.27 ± 17.90 | 1.45 ± 0.23 | 91.97 ± 8.18 | 0.65 ± 0.06 | 0.12 ± 0.03 |
|  |  |  | 60–100 | 54.33 ± 1.57 | 0.20 ± 0.02 | 0.21 ± 0.01 | 357.89 ± 17.90 | 1.88 ± 0.23 | 270.58 ± 8.18 | 1.73 ± 0.06 | 0.11 ± 0.03 |
|  |  | Drought | 0–30 | 31.89 ± 1.57 | 0.78 ± 0.02 | 0.44 ± 0.01 | 437.80 ± 17.90 | 4.79 ± 0.23 | 41.01 ± 8.18 | 1.35 ± 0.06 | 0.16 ± 0.03 |
|  |  |  | 30–60 | 13.89 ± 1.57 | 0.16 ± 0.02 | 0.31 ± 0.01 | 134.42 ± 17.90 | 1.04 ± 0.23 | 86.19 ± 8.18 | 0.59 ± 0.06 | 0.16 ± 0.03 |
|  |  |  | 60–100 | 25.46 ± 1.57 | 0.10 ± 0.02 | 0.20 ± 0.01 | 158.20 ± 17.90 | 0.78 ± 0.23 | 251.09 ± 8.18 | 0.81 ± 0.06 | 0.13 ± 0.03 |
|  | Patnos 1-4 | Control | 0–30 | 33.64 ± 1.57 | 1.00 ± 0.02 | 0.47 ± 0.01 | 495.02 ± 17.90 | 5.80 ± 0.23 | 33.64 ± 8.18 | 1.43 ± 0.06 | 0.17 ± 0.03 |
|  |  |  | 30–60 | 9.88 ± 1.57 | 0.15 ± 0.02 | 0.31 ± 0.01 | 94.90 ± 17.90 | 0.73 ± 0.23 | 65.39 ± 8.18 | 0.42 ± 0.06 | 0.21 ± 0.03 |
|  |  |  | 60–100 | 27.96 ± 1.57 | 0.16 ± 0.02 | 0.20 ± 0.01 | 176.08 ± 17.90 | 0.89 ± 0.23 | 176.14 ± 8.18 | 0.89 ± 0.06 | 0.19 ± 0.03 |
|  |  | Drought | 0–30 | 18.74 ± 1.57 | 0.54 ± 0.02 | 0.48 ± 0.01 | 280.84 ± 17.90 | 3.36 ± 0.23 | 35.01 ± 8.18 | 0.80 ± 0.06 | 0.16 ± 0.03 |
|  |  |  | 30–60 | 13.44 ± 1.57 | 0.17 ± 0.02 | 0.31 ± 0.01 | 130.69 ± 17.90 | 1.01 ± 0.23 | 77.58 ± 8.18 | 0.57 ± 0.06 | 0.17 ± 0.03 |
|  |  |  | 60–100 | 26.99 ± 1.57 | 0.12 ± 0.02 | 0.18 ± 0.01 | 151.07 ± 17.90 | 0.67 ± 0.23 | 220.89 ± 8.18 | 0.86 ± 0.06 | 0.18 ± 0.03 |
|  | Saray 2-5 | Control | 0–30 | 39.72 ± 1.57 | 1.32 ± 0.02 | 0.44 ± 0.01 | 545.20 ± 17.90 | 5.96 ± 0.23 | 30.09 ± 8.18 | 1.69 ± 0.06 | 0.22 ± 0.03 |
|  |  |  | 30–60 | 27.50 ± 1.57 | 0.30 ± 0.02 | 0.34 ± 0.01 | 292.17 ± 17.90 | 2.49 ± 0.23 | 93.18 ± 8.18 | 1.17 ± 0.06 | 0.13 ± 0.03 |
|  |  |  | 60–100 | 35.36 ± 1.57 | 0.33 ± 0.02 | 0.24 ± 0.01 | 260.79 ± 17.90 | 1.53 ± 0.23 | 107.16 ± 8.18 | 1.13 ± 0.06 | 0.23 ± 0.03 |
|  |  | Drought | 0–30 | 37.43 ± 1.57 | 0.98 ± 0.02 | 0.45 ± 0.01 | 532.75 ± 17.90 | 6.04 ± 0.23 | 38.36 ± 8.18 | 1.59 ± 0.06 | 0.16 ± 0.03 |
|  |  |  | 30–60 | 18.93 ± 1.57 | 0.15 ± 0.02 | 0.34 ± 0.01 | 200.75 ± 17.90 | 1.71 ± 0.23 | 129.13 ± 8.18 | 0.80 ± 0.06 | 0.09 ± 0.03 |
|  |  |  | 60–100 | 32.19 ± 1.57 | 0.18 ± 0.02 | 0.23 ± 0.01 | 235.66 ± 17.90 | 1.37 ± 0.23 | 183.45 ± 8.18 | 1.03 ± 0.06 | 0.13 ± 0.03 |

SA: surface area (cm²); Vol: volume (cm³); SRL: specific root length (m g⁻¹); RLD: root length density (m cm⁻³); RTD: root tissue density (g cm⁻³). Values are mean ± SE (n = 5).

**Supplementary Table S4.** Shoot, yield, physiological and whole-root traits (mean ± SE) across genotypes, water treatments, and years.

**S4a. Morphological and growth-related traits**

| **Year** | **Genotype** | **Water** | **Plant Height (cm)** | **Spike Length (cm)** | **Fertile Tillers** | **Sterile Tillers** | **Heading Time** | **Maturity Time** |
| --- | --- | --- | --- | --- | --- | --- | --- | --- |
| 1 | Bitlis 1-4 | Control | 99.36 ± 1.60 | 11.22 ± 0.15 | 16.40 ± 0.22 | 1.34 ± 0.11 | 52.80 ± 0.28 | 127.60 ± 0.32 |
|  |  | Drought | 82.94 ± 1.60 | 10.60 ± 0.15 | 14.96 ± 0.22 | 1.66 ± 0.11 | 53.00 ± 0.28 | 117.40 ± 0.32 |
|  | Bitlis 9-1 | Control | 98.58 ± 1.60 | 6.98 ± 0.15 | 14.76 ± 0.22 | 0.60 ± 0.11 | 60.80 ± 0.28 | 141.20 ± 0.32 |
|  |  | Drought | 80.42 ± 1.60 | 6.54 ± 0.15 | 13.44 ± 0.22 | 1.92 ± 0.11 | 60.80 ± 0.28 | 135.60 ± 0.32 |
|  | Erciş 5-6 | Control | 93.10 ± 1.60 | 9.62 ± 0.15 | 14.12 ± 0.22 | 1.86 ± 0.11 | 50.80 ± 0.28 | 124.40 ± 0.32 |
|  |  | Drought | 85.94 ± 1.60 | 9.90 ± 0.15 | 13.42 ± 0.22 | 2.92 ± 0.11 | 48.60 ± 0.28 | 120.80 ± 0.32 |
|  | Erciş 8-2 | Control | 111.98 ± 1.60 | 10.74 ± 0.15 | 14.62 ± 0.22 | 2.46 ± 0.11 | 64.20 ± 0.28 | 168.00 ± 0.32 |
|  |  | Drought | 82.30 ± 1.60 | 9.22 ± 0.15 | 13.38 ± 0.22 | 3.52 ± 0.11 | 64.40 ± 0.28 | 151.00 ± 0.32 |
|  | Gerek-79 | Control | 76.30 ± 1.60 | 8.80 ± 0.15 | 14.88 ± 0.22 | 1.56 ± 0.11 | 52.40 ± 0.28 | 134.40 ± 0.32 |
|  |  | Drought | 70.70 ± 1.60 | 8.02 ± 0.15 | 13.06 ± 0.22 | 2.22 ± 0.11 | 52.80 ± 0.28 | 124.80 ± 0.32 |
|  | Karasu | Control | 95.82 ± 1.60 | 11.22 ± 0.15 | 16.24 ± 0.22 | 2.02 ± 0.11 | 65.20 ± 0.28 | 141.60 ± 0.32 |
|  |  | Drought | 76.33 ± 1.60 | 10.16 ± 0.15 | 13.78 ± 0.22 | 3.50 ± 0.11 | 64.80 ± 0.28 | 126.20 ± 0.32 |
|  | Muradiye 12-2 | Control | 92.94 ± 1.60 | 10.56 ± 0.15 | 15.38 ± 0.22 | 2.52 ± 0.11 | 45.40 ± 0.28 | 125.80 ± 0.32 |
|  |  | Drought | 75.78 ± 1.60 | 9.86 ± 0.15 | 13.12 ± 0.22 | 3.46 ± 0.11 | 45.40 ± 0.28 | 119.20 ± 0.32 |
|  | Patnos 1-4 | Control | 104.20 ± 1.60 | 9.94 ± 0.15 | 16.10 ± 0.22 | 2.26 ± 0.11 | 54.60 ± 0.28 | 154.00 ± 0.32 |
|  |  | Drought | 83.16 ± 1.60 | 9.04 ± 0.15 | 14.36 ± 0.22 | 3.48 ± 0.11 | 54.80 ± 0.28 | 146.20 ± 0.32 |
|  | Saray 2-5 | Control | 98.40 ± 1.60 | 8.88 ± 0.15 | 12.74 ± 0.22 | 2.48 ± 0.11 | 75.00 ± 0.28 | 154.60 ± 0.32 |
|  |  | Drought | 83.50 ± 1.60 | 9.72 ± 0.15 | 12.54 ± 0.22 | 4.62 ± 0.11 | 75.80 ± 0.28 | 144.40 ± 0.32 |
| 2 | Bitlis 1-4 | Control | 102.20 ± 1.60 | 11.38 ± 0.15 | 16.76 ± 0.22 | 1.10 ± 0.11 | 42.60 ± 0.28 | 116.40 ± 0.32 |
|  |  | Drought | 86.04 ± 1.60 | 10.82 ± 0.15 | 14.93 ± 0.22 | 1.62 ± 0.11 | 42.60 ± 0.28 | 108.60 ± 0.32 |
|  | Bitlis 9-1 | Control | 88.68 ± 1.60 | 7.44 ± 0.15 | 15.24 ± 0.22 | 1.16 ± 0.11 | 60.60 ± 0.28 | 158.60 ± 0.32 |
|  |  | Drought | 75.78 ± 1.60 | 7.10 ± 0.15 | 14.18 ± 0.22 | 2.24 ± 0.11 | 60.60 ± 0.28 | 151.40 ± 0.32 |
|  | Erciş 5-6 | Control | 95.38 ± 1.60 | 10.20 ± 0.15 | 15.16 ± 0.22 | 1.72 ± 0.11 | 42.20 ± 0.28 | 123.20 ± 0.32 |
|  |  | Drought | 91.18 ± 1.60 | 9.96 ± 0.15 | 13.92 ± 0.22 | 2.88 ± 0.11 | 41.80 ± 0.28 | 117.60 ± 0.32 |
|  | Erciş 8-2 | Control | 103.60 ± 1.60 | 10.60 ± 0.15 | 13.64 ± 0.22 | 1.72 ± 0.11 | 65.00 ± 0.28 | 166.80 ± 0.32 |
|  |  | Drought | 92.22 ± 1.60 | 9.34 ± 0.15 | 14.24 ± 0.22 | 2.82 ± 0.11 | 64.60 ± 0.28 | 154.40 ± 0.32 |
|  | Gerek-79 | Control | 88.72 ± 1.60 | 9.34 ± 0.15 | 15.00 ± 0.22 | 1.52 ± 0.11 | 46.60 ± 0.28 | 121.80 ± 0.32 |
|  |  | Drought | 82.70 ± 1.60 | 8.34 ± 0.15 | 14.22 ± 0.22 | 2.02 ± 0.11 | 46.40 ± 0.28 | 114.40 ± 0.32 |
|  | Karasu | Control | 103.16 ± 1.60 | 11.44 ± 0.15 | 16.18 ± 0.22 | 1.76 ± 0.11 | 71.40 ± 0.28 | 152.20 ± 0.32 |
|  |  | Drought | 89.12 ± 1.60 | 10.20 ± 0.15 | 14.28 ± 0.22 | 2.62 ± 0.11 | 71.20 ± 0.28 | 136.20 ± 0.32 |
|  | Muradiye 12-2 | Control | 99.22 ± 1.60 | 10.78 ± 0.15 | 15.64 ± 0.22 | 2.24 ± 0.11 | 44.40 ± 0.28 | 121.60 ± 0.32 |
|  |  | Drought | 80.00 ± 1.60 | 10.50 ± 0.15 | 14.66 ± 0.22 | 2.74 ± 0.11 | 44.40 ± 0.28 | 114.20 ± 0.32 |
|  | Patnos 1-4 | Control | 112.46 ± 1.60 | 10.46 ± 0.15 | 15.48 ± 0.22 | 2.24 ± 0.11 | 58.20 ± 0.28 | 159.60 ± 0.32 |
|  |  | Drought | 98.00 ± 1.60 | 9.46 ± 0.15 | 14.54 ± 0.22 | 4.00 ± 0.11 | 58.40 ± 0.28 | 148.40 ± 0.32 |
|  | Saray 2-5 | Control | 100.86 ± 1.60 | 10.30 ± 0.15 | 15.86 ± 0.22 | 2.08 ± 0.11 | 80.60 ± 0.28 | 160.80 ± 0.32 |
|  |  | Drought | 91.06 ± 1.60 | 9.78 ± 0.15 | 14.40 ± 0.22 | 3.41 ± 0.11 | 80.60 ± 0.28 | 151.80 ± 0.32 |

**S4b. Yield, physiological and whole-root traits**

| **Grain Yield (g)** | **Biological Yield (g)** | **Stem Number** | **SPAD** | **Shoot Dry Weight (g)** | **Root Length (cm)** | **Root Volume (cm³)** | **Root Surface Area (cm²)** | **Root Dry Weight (g)** |
| --- | --- | --- | --- | --- | --- | --- | --- | --- |
| 15.48 ± 0.19 | 34.30 ± 0.58 | 6.40 ± 0.23 | 45.20 ± 1.05 | 5.60 ± 0.38 | 102.50 ± 1.58 | 12.40 ± 0.53 | 142.10 ± 2.45 | 2.15 ± 0.16 |
| 9.78 ± 0.19 | 24.04 ± 0.58 | 4.80 ± 0.23 | 39.10 ± 1.05 | 4.20 ± 0.38 | 125.40 ± 1.58 | 15.80 ± 0.53 | 168.30 ± 2.45 | 3.00 ± 0.16 |
| 8.50 ± 0.19 | 24.02 ± 0.58 | 6.10 ± 0.23 | 46.80 ± 1.05 | 5.10 ± 0.38 | 96.20 ± 1.58 | 11.20 ± 0.53 | 134.50 ± 2.45 | 1.85 ± 0.16 |
| 6.74 ± 0.19 | 22.32 ± 0.58 | 4.50 ± 0.23 | 40.20 ± 1.05 | 3.90 ± 0.38 | 115.30 ± 1.58 | 14.10 ± 0.53 | 156.40 ± 2.45 | 2.65 ± 0.16 |
| 11.00 ± 0.19 | 33.90 ± 0.58 | 5.70 ± 0.23 | 43.50 ± 1.05 | 4.50 ± 0.38 | 105.40 ± 1.58 | 12.80 ± 0.53 | 148.60 ± 2.45 | 2.40 ± 0.16 |
| 8.28 ± 0.19 | 21.14 ± 0.58 | 4.10 ± 0.23 | 37.40 ± 1.05 | 3.50 ± 0.38 | 128.60 ± 1.58 | 16.90 ± 0.53 | 176.20 ± 2.45 | 3.25 ± 0.16 |
| 11.90 ± 0.19 | 32.12 ± 0.58 | 6.90 ± 0.23 | 49.10 ± 1.05 | 5.90 ± 0.38 | 112.50 ± 1.58 | 14.10 ± 0.53 | 160.40 ± 2.45 | 2.70 ± 0.16 |
| 6.35 ± 0.19 | 22.96 ± 0.58 | 5.30 ± 0.23 | 42.80 ± 1.05 | 4.60 ± 0.38 | 138.40 ± 1.58 | 18.50 ± 0.53 | 192.60 ± 2.45 | 3.80 ± 0.16 |
| 9.56 ± 0.19 | 20.44 ± 0.58 | 5.50 ± 0.23 | 43.60 ± 1.05 | 4.30 ± 0.38 | 90.20 ± 1.58 | 10.10 ± 0.53 | 125.40 ± 2.45 | 1.65 ± 0.16 |
| 6.99 ± 0.19 | 19.72 ± 0.58 | 3.90 ± 0.23 | 36.20 ± 1.05 | 3.20 ± 0.38 | 107.50 ± 1.58 | 13.00 ± 0.53 | 144.20 ± 2.45 | 2.30 ± 0.16 |
| 12.24 ± 0.19 | 32.30 ± 0.58 | 7.30 ± 0.23 | 51.20 ± 1.05 | 6.40 ± 0.38 | 119.80 ± 1.58 | 15.30 ± 0.53 | 170.80 ± 2.45 | 3.05 ± 0.16 |
| 6.44 ± 0.19 | 21.48 ± 0.58 | 5.70 ± 0.23 | 45.40 ± 1.05 | 5.00 ± 0.38 | 147.60 ± 1.58 | 19.80 ± 0.53 | 206.50 ± 2.45 | 4.15 ± 0.16 |
| 9.04 ± 0.19 | 28.06 ± 0.58 | 6.30 ± 0.23 | 46.10 ± 1.05 | 5.30 ± 0.38 | 100.40 ± 1.58 | 11.90 ± 0.53 | 139.20 ± 2.45 | 2.05 ± 0.16 |
| 5.08 ± 0.19 | 18.74 ± 0.58 | 4.70 ± 0.23 | 39.80 ± 1.05 | 4.00 ± 0.38 | 122.10 ± 1.58 | 15.10 ± 0.53 | 164.30 ± 2.45 | 2.80 ± 0.16 |
| 8.68 ± 0.19 | 23.90 ± 0.58 | 6.70 ± 0.23 | 48.50 ± 1.05 | 5.70 ± 0.38 | 109.20 ± 1.58 | 13.40 ± 0.53 | 154.50 ± 2.45 | 2.55 ± 0.16 |
| 3.99 ± 0.19 | 15.92 ± 0.58 | 5.10 ± 0.23 | 42.10 ± 1.05 | 4.40 ± 0.38 | 134.50 ± 1.58 | 17.80 ± 0.53 | 182.60 ± 2.45 | 3.60 ± 0.16 |
| 10.85 ± 0.19 | 30.18 ± 0.58 | 7.10 ± 0.23 | 50.10 ± 1.05 | 6.10 ± 0.38 | 115.40 ± 1.58 | 14.80 ± 0.53 | 165.20 ± 2.45 | 2.90 ± 0.16 |
| 4.66 ± 0.19 | 18.34 ± 0.58 | 5.50 ± 0.23 | 44.00 ± 1.05 | 4.80 ± 0.38 | 142.30 ± 1.58 | 19.10 ± 0.53 | 199.80 ± 2.45 | 3.95 ± 0.16 |
| 19.30 ± 0.19 | 44.62 ± 0.58 | 7.20 ± 0.23 | 47.96 ± 1.05 | 6.20 ± 0.38 | 105.90 ± 1.58 | 13.20 ± 0.53 | 149.10 ± 2.45 | 2.45 ± 0.16 |
| 12.30 ± 0.19 | 30.80 ± 0.58 | 5.60 ± 0.23 | 41.38 ± 1.05 | 4.88 ± 0.38 | 131.80 ± 1.58 | 17.00 ± 0.53 | 176.50 ± 2.45 | 3.28 ± 0.16 |
| 10.90 ± 0.19 | 45.24 ± 0.58 | 6.70 ± 0.23 | 49.60 ± 1.05 | 5.86 ± 0.38 | 100.60 ± 1.58 | 11.80 ± 0.53 | 141.90 ± 2.45 | 2.11 ± 0.16 |
| 8.18 ± 0.19 | 31.90 ± 0.58 | 5.10 ± 0.23 | 42.80 ± 1.05 | 4.54 ± 0.38 | 121.10 ± 1.58 | 15.50 ± 0.53 | 165.20 ± 2.45 | 2.95 ± 0.16 |
| 11.60 ± 0.19 | 37.22 ± 0.58 | 6.30 ± 0.23 | 46.82 ± 1.05 | 5.22 ± 0.38 | 111.80 ± 1.58 | 13.60 ± 0.53 | 156.20 ± 2.45 | 2.68 ± 0.16 |
| 9.36 ± 0.19 | 23.46 ± 0.58 | 4.70 ± 0.23 | 39.80 ± 1.05 | 4.10 ± 0.38 | 136.20 ± 1.58 | 18.10 ± 0.53 | 187.00 ± 2.45 | 3.59 ± 0.16 |
| 14.76 ± 0.19 | 52.68 ± 0.58 | 7.50 ± 0.23 | 51.70 ± 1.05 | 6.58 ± 0.38 | 119.10 ± 1.58 | 15.10 ± 0.53 | 169.20 ± 2.45 | 3.06 ± 0.16 |
| 8.42 ± 0.19 | 36.84 ± 0.58 | 5.90 ± 0.23 | 45.60 ± 1.05 | 5.32 ± 0.38 | 146.80 ± 1.58 | 19.90 ± 0.53 | 204.40 ± 2.45 | 4.12 ± 0.16 |
| 11.52 ± 0.19 | 30.66 ± 0.58 | 6.10 ± 0.23 | 46.00 ± 1.05 | 4.90 ± 0.38 | 94.80 ± 1.58 | 10.70 ± 0.53 | 131.80 ± 2.45 | 1.87 ± 0.16 |
| 8.86 ± 0.19 | 28.32 ± 0.58 | 4.50 ± 0.23 | 38.80 ± 1.05 | 3.90 ± 0.38 | 114.10 ± 1.58 | 14.20 ± 0.53 | 152.60 ± 2.45 | 2.60 ± 0.16 |
| 17.86 ± 0.19 | 41.60 ± 0.58 | 7.90 ± 0.23 | 54.00 ± 1.05 | 7.20 ± 0.38 | 125.00 ± 1.58 | 16.30 ± 0.53 | 179.60 ± 2.45 | 3.35 ± 0.16 |
| 11.74 ± 0.19 | 35.68 ± 0.58 | 6.30 ± 0.23 | 48.20 ± 1.05 | 5.80 ± 0.38 | 154.80 ± 1.58 | 21.40 ± 0.53 | 218.30 ± 2.45 | 4.55 ± 0.16 |
| 9.88 ± 0.19 | 30.26 ± 0.58 | 6.90 ± 0.23 | 48.70 ± 1.05 | 6.00 ± 0.38 | 105.20 ± 1.58 | 12.50 ± 0.53 | 146.40 ± 2.45 | 2.25 ± 0.16 |
| 6.18 ± 0.19 | 25.10 ± 0.58 | 5.30 ± 0.23 | 42.60 ± 1.05 | 4.70 ± 0.38 | 128.70 ± 1.58 | 16.10 ± 0.53 | 172.70 ± 2.45 | 3.10 ± 0.16 |
| 11.90 ± 0.19 | 34.16 ± 0.58 | 7.30 ± 0.23 | 51.10 ± 1.05 | 6.50 ± 0.38 | 115.80 ± 1.58 | 14.20 ± 0.53 | 162.70 ± 2.45 | 2.85 ± 0.16 |
| 7.80 ± 0.19 | 25.48 ± 0.58 | 5.70 ± 0.23 | 44.90 ± 1.05 | 5.20 ± 0.38 | 141.90 ± 1.58 | 19.00 ± 0.53 | 193.80 ± 2.45 | 3.90 ± 0.16 |
| 14.24 ± 0.19 | 41.86 ± 0.58 | 7.70 ± 0.23 | 52.90 ± 1.05 | 7.00 ± 0.38 | 121.80 ± 1.58 | 15.60 ± 0.53 | 173.60 ± 2.45 | 3.20 ± 0.16 |
| 9.10 ± 0.19 | 31.26 ± 0.58 | 6.10 ± 0.23 | 46.80 ± 1.05 | 5.50 ± 0.38 | 151.30 ± 1.58 | 20.50 ± 0.53 | 211.40 ± 2.45 | 4.35 ± 0.16 |

Values are mean ± SE (n = 5). Year 1 = 2021; Year 2 = 2022.

**Supplementary Table S5.** Pearson correlation coefficients among shoot traits and yield components.

| **Trait** | **Plant Height** | **Spike Length** | **Fertile Spike** | **Sterile Spike** | **Heading** | **Maturity** | **Grain Yield** | **Biological Yield** | **H.I.** | **Tillers** | **Spike Weight** | **BTS** | **BTA** | **SPAD** | **Senescence** |
| --- | --- | --- | --- | --- | --- | --- | --- | --- | --- | --- | --- | --- | --- | --- | --- |
| Plant Height | 1 |  |  |  |  |  |  |  |  |  |  |  |  |  |  |
| Spike Length | 0.454** | 1 |  |  |  |  |  |  |  |  |  |  |  |  |  |
| Fertile Spike | 0.512** | 0.438** | 1 |  |  |  |  |  |  |  |  |  |  |  |  |
| Sterile Spike | −0.271** | 0.064 | −0.514** | 1 |  |  |  |  |  |  |  |  |  |  |  |
| Heading | 0.177* | −0.094 | −0.166* | 0.261** | 1 |  |  |  |  |  |  |  |  |  |  |
| Maturity | 0.446** | −0.137 | −0.008 | 0.120 | 0.756** | 1 |  |  |  |  |  |  |  |  |  |
| Grain Yield | 0.611** | 0.494** | 0.606** | −0.611** | 0.045 | 0.102 | 1 |  |  |  |  |  |  |  |  |
| Biological Yield | 0.551** | 0.292** | 0.434** | −0.481** | 0.201** | 0.345** | 0.809** | 1 |  |  |  |  |  |  |  |
| Harvest Index | 0.264** | 0.361** | 0.406** | −0.470** | −0.219** | −0.271** | 0.584** | 0.028 | 1 |  |  |  |  |  |  |
| Tillers | 0.297** | 0.259** | 0.156* | −0.037 | 0.057 | 0.228** | 0.294** | 0.578** | −0.271** | 1 |  |  |  |  |  |
| Spike Weight | 0.264** | 0.007 | 0.331** | −0.193** | 0.030 | 0.235** | 0.223** | 0.281** | −0.012 | −0.041 | 1 |  |  |  |  |
| BTS | 0.255** | 0.382** | 0.165* | −0.168* | −0.043 | −0.042 | 0.354** | 0.310** | 0.210** | 0.161* | −0.069 | 1 |  |  |  |
| BTA | 0.135 | −0.257** | −0.052 | −0.101 | −0.137 | −0.021 | −0.078 | 0.006 | −0.113 | 0.277** | −0.199** | −0.092 | 1 |  |  |
| SPAD | −0.068 | 0.361** | 0.225** | −0.405** | −0.346** | −0.533** | 0.414** | 0.259** | 0.369** | 0.147* | −0.213** | 0.247** | −0.091 | 1 |  |
| Senescence | −0.211** | −0.381** | −0.233** | 0.480** | 0.441** | 0.384** | −0.446** | −0.213** | −0.557** | −0.001 | 0.220** | −0.439** | −0.058 | −0.656** | 1 |

** p < 0.01; * p < 0.05. BTS: grains per spike; BTA: grain weight per spike; H.I.: harvest index.

**Supplementary Table S6.** Pearson correlation coefficients among root traits.

| **Trait** | **Total Root Length** | **Total Root Dry Weight** | **Total Root Surface Area** | **Total Root Volume** | **Deep Root Index** | **Root Fraction 60–100 cm** | **Total SRL** | **Total RTD** | **Total RLD** | **Root/Shoot Ratio** |
| --- | --- | --- | --- | --- | --- | --- | --- | --- | --- | --- |
| Total Root Length | 1 |  |  |  |  |  |  |  |  |  |
| Total Root Dry Weight | 0.590** | 1 |  |  |  |  |  |  |  |  |
| Total Root Surface Area | 0.938** | 0.604** | 1 |  |  |  |  |  |  |  |
| Total Root Volume | 0.741** | 0.515** | 0.921** | 1 |  |  |  |  |  |  |
| Deep Root Index | 0.542** | 0.175* | 0.350** | 0.177* | 1 |  |  |  |  |  |
| Root Fraction 60–100 cm | 0.542** | 0.175* | 0.350** | 0.177* | 1.000** | 1 |  |  |  |  |
| Total SRL | 0.486** | −0.389** | 0.397** | 0.266** | 0.437** | 0.437** | 1 |  |  |  |
| Total RTD | −0.087 | 0.485** | −0.260** | −0.437** | 0.024 | 0.024 | −0.588** | 1 |  |  |
| Total RLD | 1.000** | 0.590** | 0.938** | 0.741** | 0.542** | 0.542** | 0.486** | −0.087 | 1 |  |
| Root/Shoot Ratio | 0.209** | 0.443** | 0.264** | 0.355** | 0.274** | 0.274** | −0.221** | 0.130 | 0.209** | 1 |

** p < 0.01; * p < 0.05. SRL: specific root length; RTD: root tissue density; RLD: root length density.

**Supplementary Table S7.** Integrated drought response indices of wheat genotypes based on yield retention, biomass retention, and root trait responses.

| **Genotype** | **Yield Retention (%)** | **Biomass Retention (%)** | **Root Length Response (%)** | **Deep Root Response (%)** |
| --- | --- | --- | --- | --- |
| Bitlis 1-4 | 63.2 | 69.4 | −10.6 | +33.4 |
| Bitlis 9-1 | 77.3 | 78.3 | −39.4 | −27.5 |
| Erciş 5-6 | 77.9 | 62.6 | +19.2 | +44.2 |
| Erciş 8-2 | 55.6 | 70.5 | −22.6 | −5.3 |
| Gerek-79 | 75.2 | 93.7 | −28.8 | −29.9 |
| Karasu | 60.3 | 77.3 | +11.7 | +13.7 |
| Muradiye 12-2 | 58.9 | 75.0 | −17.7 | −13.6 |
| Patnos 1-4 | 57.3 | 71.4 | −14.1 | +9.5 |
| Saray 2-5 | 55.2 | 68.9 | −8.9 | +0.8 |

Yield Retention and Biomass Retention are expressed as percentage of control value maintained under drought. Root Length Response and Deep Root Response are expressed as percentage change relative to control.

**Supplementary Table S8.** Principal component loadings (unrotated PCA) for yield components and root traits based on standardized values from 180 observations.

| **Trait** | **PC1 (44.8%)** | **PC2 (27.7%)** |
| --- | --- | --- |
| Grain Yield | 0.621 | −0.297 |
| Biological Yield | 0.534 | −0.246 |
| Harvest Index | 0.336 | −0.147 |
| Total Root Length | 0.425 | 0.528 |
| Deep Root Index | 0.190 | 0.742 |

PC1 and PC2 collectively explained 72.5% of the total variation. Higher absolute loadings indicate stronger association with the respective principal component.
